# Comprehensive Analysis of Genic Male Sterility-Related Genes in *Brassica* *rapa* Using a Newly Developed Br300K Oligomeric Chip

**DOI:** 10.1371/journal.pone.0072178

**Published:** 2013-09-11

**Authors:** Xiangshu Dong, Hui Feng, Ming Xu, Jeongyeo Lee, Yeon Ki Kim, Yong Pyo Lim, Zhongyun Piao, Young Doo Park, Hong Ma, Yoonkang Hur

**Affiliations:** 1 Department of Biological Sciences, Chungnam National University, Daejeon, Korea; 2 Department of Horticulture, Shenyang Agricultural University, Shenyang, China; 3 GreenGene Biotech Inc, Genomics and Genetics Institute, Yongin, Korea; 4 Department of Horticulture, Chungnam National University, Daejeon, Korea; 5 Department of Horticultural Biotechnology, Kyung Hee University, Yongin, Korea; 6 State Key Laboratory of Genetic Engineering, Institute of Plant Biology, Center for Evolutionary Biology, School of Life Sciences, Fudan University, Shanghai, China; Nanjing Agricultural University, China

## Abstract

To identify genes associated with genic male sterility (GMS) that could be useful for hybrid breeding in Chinese cabbage ( *Brassica* *rapa* ssp. *pekinensis* ), floral bud transcriptome analysis was carried out using a *B* *. rapa* microarray with 300,000 probes (Br300K). Among 47,548 clones deposited on a Br300K microarray with seven probes of 60 nt length within the 3' 150 bp region, a total of 10,622 genes were differentially expressed between fertile and sterile floral buds; 4,774 and 5,848 genes were up-regulated over 2-fold in fertile and sterile buds, respectively. However, the expression of 1,413 and 199 genes showed fertile and sterile bud-specific features, respectively. Genes expressed specifically in fertile buds, possibly GMS-related genes, included homologs of several *Arabidopsis* male sterility-related genes, genes associated with the cell wall and synthesis of its surface proteins, pollen wall and coat components, signaling components, and nutrient supplies. However, most early genes for pollen development, genes for primexine and callose formation, and genes for pollen maturation and anther dehiscence showed no difference in expression between fertile and sterile buds. Some of the known genes associated with *Arabidopsis* pollen development showed similar expression patterns to those seen in this study, while others did not. *BrbHLH89* and *BrMYP99* are putative GMS genes. Additionally, 17 novel genes identified only in *B* *. rapa* were specifically and highly expressed only in fertile buds, implying the possible involvement in male fertility. All data suggest that Chinese cabbage GMS might be controlled by genes acting in post-meiotic tapetal development that are different from those known to be associated with *Arabidopsis* male sterility.

## Introduction

Pollen development, a process stemming from anther cell division and differentiation leading to male meiosis, as well as pollen wall and coat development and anther dehiscence, relies on the functions of numerous genes from both the microspore itself and sporophytic anther tissues including the tapetum [1–7]. Since pollen development is known to be regulated by the levels of transcripts and small RNAs [8], transcriptome analysis can provide insights into male sterility. During the last decade, transcriptomic studies of the anther have identified thousands of transcripts expressed in various plant species, including *B. oleracea* [9]. In the model plant *Arabidopsis* , gene expression profile studies by microarray during pollen development have been extensively carried out to identify genes specific for stamen [10–14] and pollen development [15–20]. Since the *Brassica* and *Arabidopsis* genera share about 85% exon sequence similarity [21], the *Arabidopsis* microarray was applied to *Brassica* species [22] to investigate gene expression in flower buds of the *Ms-cd1* (male sterile mutants of *B. oleracea*) [23] and in male sterility in *B. napus* [24,25]. However, these arrays represent parts of genes for each plant, and do not cover the majority of genes. Using a *B* *. rapa* -specific microarray, transcriptome analysis from floral buds, which include both gametophytic and sporophytic tissues, was conducted to identify genes associated with genic male sterility (GMS) in Chinese cabbage.

In *Arabidopsis* , several core genes controlling anther and pollen development have been uncovered by molecular genetic studies [6,14,26–28]. At an early anther stage, *SPL/NZZ* (*SPROROCYTELESS/NOZZLE*) is required for sporocyte formation and anther cell division [29–31]. *EMS1/EXS* (*EXCESS MICROSPOROCYTES 1/EXTRA SPOROGENOUS CELLS*) is essential for tapetum formation and differentiation [32–34]. Tapetal function and pollen development are then controlled by several transcription factor genes in a sequential and overlapping manner. These include: *DYT1* (*DYSFUNCTIONAL TAPETUM1*), controlling an early tapetal developmental stage [35]; TDF1 (Tapetal Development and Function 1), controlling callose dissolution around microspores and exine formation of the pollen wall [36]; and *AMS* (*ABORTED MICROSPORES*), *MS1* (*MALE STERILITY 1*), and *MYB103/80*, controlling post-meiotic tapetal function and pollen development [28,35]. *AtMYB103, MS1*, and *AMS* also influence programmed cell death (PCD) in the tapetum after microspore mitosis I [20,37–39]. Many other genes, such as lipid transfer protein family genes, oleosin genes, genes associated with the phenylpropanoid and brassinosteroid biosynthesis pathways*, MS2, FLP1* (*Faceless Pollen-1*)*, DEX1* (*Defective in Exine Pattern Formation*), and NEF1 (No Exine Formation 1), are involved in late steps of pollen development [28,40].

Chinese cabbage ( *Brassica* *rapa* L. *ssp.* *pekinensis* ), a popular leafy vegetable, is a cross-pollinating crop with significant heterosis; however, F_1_ seed production using manual pollination is limited by the small reproductive organ and small number of seeds per fruit. Therefore, the method of choice to date is to use self-incompatible lines or male sterile lines. Because the utilization of self-incompatible lines is hampered by difficulty in parent reproduction, inbred depression after selfing for multiple generations, and contamination with non-hybrid seed production, the use of male sterile lines appears to be a more promising method for hybrid seed production in Chinese cabbage. In Chinese cabbage, two types of male sterile sources are available: GMS and cytoplasmic male sterility (CMS) [41]. F_1_ hybrid seeds using CMS lines have not been widely used because the F_1_ plants do not show heterosis, but rather chlorosis (a cytoplasmic negative effect), at low temperatures. By contrast, GMS has more obvious advantages, such as stable and complete sterility, extensive distribution of restorers, and no negative cytoplasmic effect; thus it has been considered to be a good male sterile resource.

Previously, Feng et al [42,43] had obtained four 100% male sterile lines in Chinese cabbage by mutual crossing of nine AB lines. They found that male sterility was controlled by three alleles at one locus: “Ms^f^” as the dominant restorer, “Ms” as the dominant sterile allele, and “ms” as the recessive fertile allele. The dominance relationship is “Ms^f^” > “Ms” > “ms”, as described in a genetic model shown in Figure S1. Although the 100% male sterile GMS line has been utilized in commercial Chinese cabbage hybrid seed production in China, molecular genetics mechanisms of GMS are totally unknown. To identify Ms^f^ gene(s), and understand GMS mechanisms in Chinese cabbage, we carried out microarray experiments using the newly developed Br300K chip designed from 47,548 *B* *. rapa* Unigenes. The results revealed that the Chinese cabbage GMS mechanism might be different from the *Arabidopsis* one. Many genes regulating pollen wall and coat formation processes were specifically up-regulated in fertile line, but down-regulated in sterile line. All data analyzed in this study indicated that Chinese cabbage GMS might be controlled by genes acting in post-meiotic tapetal development.

## Materials and Methods

### Plant materials

As shown in Figure S1, fertile plants (*Ms* ^*f*^ *Ms*) and sterile plants (*MsMS*) were obtained by planting seeds from a cross between male fertile (*Ms* ^*f*^ *Ms*) and sterile (*MsMS*) plants, segregated to a 1:1 ratio. The seeds were sown and grown in a greenhouse at Chungnam National University in spring and autumn of 2009 and 2010. After flowering, *Ms* ^*f*^ *Ms* and *MsMS* plants were identified and floral buds were sampled from at least 10 plants with transcriptome profiles representing '*f*' difference, each at different developmental stages. The bud samples were divided into three and four pools for sterile and fertile buds, respectively, and stored at -70 °C until use.

### Construction of the Br300K chip

A 300k microarray chip (Br300K; version 2.0) for *B* *. rapa* designed from 47,548 *Unigenes* (Figure S2) was manufactured at NimbleGen, Inc. (http://www.nimblegen.com/) as described recently [44]. Random GC probes (40,000) were used to monitor the hybridization efficiency and four corner fiducial controls (225) were included to assist with overlaying the grid on the image. To assess the reproducibility of the microarray analysis, we repeated the experiment two or three times with independently prepared total RNAs. The normal distribution of Cy3 intensities was tested by qqline. The data were normalized and processed with cubic spline normalization using quantiles to adjust signal variations between chips and Robust Multi-Chip Analysis (RMA) using a median polish algorithm implemented in NimbleScan [45,46].

### RNA isolation and hybridization to the Br300K Microarray GeneChip

Total RNA was isolated from samples using an easy-BLUETM total RNA extraction kit (Invitrogen, NY, U.S.A.) and was then purified using an RNeasy MinEluteTM Cleanup Kit (Qiagen, Germany). For biological repeats, RNAs were extracted from two samples collected in 2009 and 2010, and subjected to microarray analysis.

For the synthesis of double-stranded cDNAs, a Superscript Double-Stranded cDNA Synthesis Kit (Invitrogen, NY, U.S.A.) was used. Briefly, 1 µl of oligo dT primer (100 µM) and 10 µl (10 µg) of total RNA were combined and denatured at 70 °C for 10 min and renatured by cooling the mixture on ice. First-strand DNA was synthesized by adding 4 µl of 5X First Strand Buffer, 2 µl of 0.1M DTT, 1 µl of 10 mM dNTP mix, and 2 µl of SuperScript enzyme and by incubating at 42 °C for 1 h. To synthesize the second strand, 91 µl of DEPC-water, 30 µl of 5X Second Strand Buffer, 3 µl of 10 mM dNTP mix, 1 µl of 10 U/µl DNA ligase, 4 µl of 10 U/µl DNA Polymerase I, and 1 µl of 2 U/µl RNase H were added to the first-strand reaction mixture and the reaction was allowed to proceed at 16 °C for 2 h. After the RNA strand was removed by RNase A (Amresco, OH, U.S.A.), the reaction mixture was clarified by phenol/chloroform extraction and then cDNA was precipitated by centrifugation at 12,000 × g after adding 16 µl of 7.5 M ammonium acetate and 326 µl of cold ethanol. For the synthesis of Cy3-labeled target DNA fragments, 1 µg of double-stranded cDNA was mixed with 40 µl (1 OD) of Cy3-9mer primers (Sigma-Aldrich, MO, U.S.A.), and denatured by heating at 98 °C for 10 min. Next, 10 µl of 50X dNTP mix (10mM each), 8 µl of deionized water, and 2 µl of Klenow fragment (50 U/µl, NEB, MA, U.S.A.) were added and the reaction mixture was incubated at 37 °C for 2 h. DNA was precipitated by centrifugation at 12,000 × g after adding 11.5 µl of 5M NaCl and 110 µl of isopropanol. Precipitated samples were rehydrated with 25 µl of water. The concentration of each sample was determined by spectrophotometry. Thirteen micrograms of DNA were used for microarray hybridization. The sample was mixed with 19.5 µl of 2X hybridization buffer (NimbleGen, WI, U.S.A.) and finalized to 39 µl with deionized water. Hybridization was performed in a MAUI chamber (Biomicro, CA, U.S.A.) at 42 °C for 16 h. After the hybridization, the microarray was removed from the MAUI Hybridization Station and immediately immersed in a shallow 250 ml Wash I solution (NimbleGen, WI, U.S.A.) at 42 °C for 10–15 sec with gentle agitation and then transferred to a second dish of Wash I and incubated for 2 min with gentle agitation. The microarray was transferred into a dish of Wash II solution and further washed in Wash III solution for 15 seconds with agitation. The microarray was dried in a centrifuge for 1 min at 500 × g and scanned using a GenePix scanner 4000B (Molecular Devices, CA, U.S.A.)

The microarray was scanned with a GenePix 4000B preset with a 5 µm resolution, for Cy3 signal. Signals were digitized and analyzed by NimbleScan (NimbleGen, U.S.A.). The grid was aligned to the image with a chip design file (NimbleGen Design File, NDF). The alignment was verified to ensure that the grid corners were overlaid on the image corners. This was further confirmed by uniformity of scores in the program. The analysis was performed in a two-part process. First, pair report files were generated in which sequence, probe, and signal intensity information for the Cy3 channel were collected. Data-based background subtraction using a local background estimator was performed to improve fold-change estimates on arrays with high background signal. The data were normalized as mentioned in the microarray construction section. The complete microarray data have been deposited in NCBI’s Gene Expression Omnibus (GSE47665).

### Gene chip data analysis

Genes with adj.P.Value or false discovery rate below 0.05 were collected and further selected for those genes with expression greater than 1 or less than -1 at at least one stage compared with expression at stage 1. Multivariate statistical tests such as clustering, principal component analysis, and multidimensional scaling were performed with Acuity 3.1 (Molecular Devices, U.S.A.). Hierarchical clustering was performed with similarity metrics based on squared Euclidean correlation and average linkage clustering was used to calculate the distance between genes.

### Comparison of *B. rapa* genes on the Br300K microarray with other known plant genes

In the *Brassica* *rapa* 300k Microarray v2.0, designed from 47,548 *Unigenes* , 31,057 cDNA/EST-supported genes were compared with the genome sequences of *B. napus,* *Arabidopsis* , and rice sequences at the amino acid levels using BLASTP analysis. The numbers of genes for the comparison were 33,410 from the *Arabidopsis* TAIR9 database, 30,192 from the rice RAP2.0 database, and 56,628 putative ORFs among 80,696 *B. napus* consensus sequences.

### Light microscopy

Sterile and fertile floral buds at different anther developmental stages were fixed in FAA (70% ethanol, 90 ml; glacial acetic acid, 5 ml; formaldehyde, 5 ml), dehydrated in a graded ethanol series (30%, 50%, 70%, 80%, 90%, 95%, 2×100%), cleared in a dimethylbenzene series (66.67% 100% ethanol + 33.33% dimethylbenzene; 50% 100% ethanol + 50% dimethylbenzene; 33.33% 100% ethanol + 66.67% dimethylbenzene; 2 × 100% dimethylbenzene), embedded in paraffin, and sectioned (8–10 µm) using a microtome. Anther transverse sections were stained in 0.5–1% safranine and 0.1–0.2% fast green. Bright-field photographs of the anther cross-sections were taken using a compound microscope (Olympus Model BH2).

### RT-PCR analysis

Total RNA (5 µg) from each sample was combined with random hexamer primers in a SuperScript first-strand cDNA synthesis system according to the manufacturer’s instructions (Invitrogen, U.S.A.). Complementary DNA was diluted 10-fold and 1 µl of the diluted cDNA was used in a 20 µl PCR mixture. RT-PCR primers are listed in Table S1 and primers for *BrACT1*, used as controls, were 5′-GTCTTGACCTTGCTGGACGTGA-3′ (forward) and 5′-CCTTTCAGGTGGTGCAACGAC-3′ (reverse). A standard PCR was performed with 5 min denaturation at 94 °C, followed by 25 cycles of 94 °C for 30 s, 55 °C for 30 s, and 72 °C for 90 s. PCR products were analyzed following electrophoresis through a 1% agarose gel.

## Results and Discussion

### Floral structure of GMS Chinese cabbage

To investigate development defects in Chinese cabbage, flowers from sterile and fertile plants were examined (Figure S3, Table S2). All floral organ measurements except pistil length and diameter were smaller in sterile flowers than in fertile flowers (significant difference: p=0.01, by T-test). However, the morphology of all of the floral organs except for the stamens was normal. In sterile flowers, the length of the stamens was greatly reduced, with shortened filaments. In addition, anthers appeared to be thin and pale white and did not bear any pollen grain. These observations imply that genes regulating the floral organ identity seemed to be normal, whereas genes for anther and pollen development were defective or expressed abnormally. Moreover, the expression of genes associated with cell growth and hormonal signaling might be altered.

### Anther development in floral buds used in microarrays

**Figure 1:**
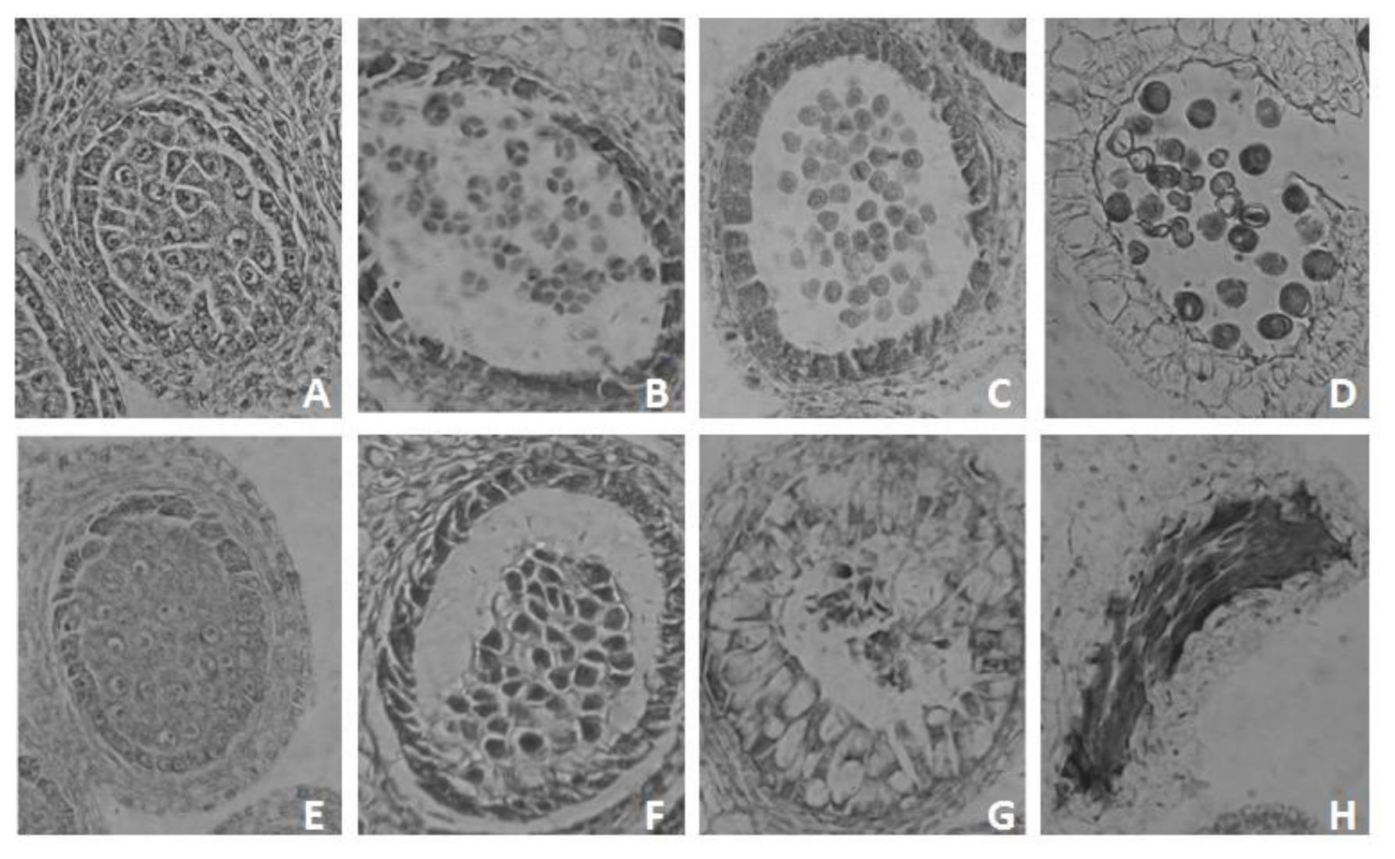
Anther development in fertile and sterile (GMS) Chinese cabbage. Chinese cabbage flower buds were fixed, embedded in paraffin, and sliced into 8–10 µm transverse sections as described in the Materials and Methods. The bud sections were stained with fast green and the counterstain safranin, and anthers were photographed by bright-field microscopy. A-D depict anther development in fertile flower buds; E-H depict anther development in sterile flower buds. A and E, microspore mother cell stage; B and F, tetrad stage; C, uninucleate microspore stage; D, mature pollen; G, abnormal tapetal cells; H, abortive pollen.

To gain information complementary to the microarray experiments, anther development was examined for sterile and fertile floral buds (Figure 1). Detailed microscopic study led to the division of anther development of Chinese cabbage into five stages: pollen mother cell (PMC), tetrad, uninucleate, bicellular, and mature pollen stages (Figure 1 plus data not shown). The anthers of sterile and fertile floral buds appeared to be similar before the tetrad stage. After the tetrad stage, the fertile anthers could release microspores, which develop into mature pollens. However, in the sterile anthers, PMCs seem to remain associated with each other in the locule, unlike the normal PMCs that dissociate from each other during meiosis. In addition, the tapetum swelled to expand at the centre of the locule. These events were followed by abnormal degradation of the endothecium and collapse of pollen grains in the mature pollen stage. Based on *Arabidopsis* microsporogenesis [28], the early microsporogenesis process should be normal in our GMS plants. Instead, genes associated with tapetal development or post-meiotic tapetal function were defective in the GMS cabbage. Taken together, the sterile buds showed two distinct defects: the failure of microspore release or imperfect tetrad formation, and the swollen tapetum layer. This may imply that expression of GMS-related genes must commence from an early stage of male sporogenesis if microspores are to be released.

Using morphological features and floral bud size, fertile and sterile bud samples were classified into four stages (F1, F2, F3, and F4) and three stages (S1, S2, and S3), respectively (Figure S4, Table 1). At each corresponding stage, the sizes of floral buds from the sterile plants were smaller than those of the fertile plants.

**Table 1:** Description of floral buds used in the microarray analysis.

**Bud samples**		**Bud size**	**Pollen developmental stage**	In Figure **1**
Sterile buds	S1	<1.5 mm	Before tetrad stage	E
	S2	1.5 mm≤ buds ≤2.5 mm	Tetrad stage	F
	S3	>2.5 mm	Aberrant pollen	G
Fertile buds	F1	<2.0 mm	Before tetrad stage	A
	F2	2.0mm≤ buds ≤2.5 mm	Tetrad stage	B
	F3	2.5mm≤ buds ≤5.0 mm	After tetrad stage, but before mature pollen	B–C
	F4	>5.0mm	Mature pollen	C–D

### Analysis of *B. rapa* genes on Br300K microarray

To demonstrate the necessity of the *B* *. rapa* microchip for Chinese cabbage study, and to verify the microarray results, genes used in construction of the Br300K chip were analyzed for sequence similarity to other plant genes. When the 31,057 *B* *. rapa* amino acid sequences with cDNA/EST supports were compared to those of *Arabidopsis* , *B. napus*, and rice, the number of genes with BLASTP scores higher than 30 were 18,078, 17,441, and 15,361, respectively. Figure S5A shows the percentage of similar genes in the three plants after grouping genes according to BLASTP score bins: <=70, 100, 200, 300, and > = 300. As expected, more *B* *. rapa* sequences showed homology with *Arabidopsis* and *B. napus* than with rice. In the BLAST score bin 300–1,000, 40.6% and 39.8% of the genes had homologs in *Arabidopsis* and *B. napus*, respectively, while 18.9% of the genes had homologs in rice. Interestingly, in the bins less than 200, more genes had counterparts in rice than in *Arabidopsis* and *B. napus*. This is consistent with the longer evolutionary distance between *B* *. rapa* and rice compared with that between *B* *. rapa* and *B. napus* or *Arabidopsis* *.*

When the probe-designed regions of *B* *. rapa* genes were compared with the 18,078 *Arabidopsis* homologs, the percentage distribution of BLASTn score bins was lower than that of BLASTP score bins (Figure S5B). Comparison of 39,181 *B* *. rapa* genes with *Arabidopsis* ones showed an average sequence identity of 89%, suggesting that existing *Arabidopsis* oligomeric chips are not appropriate for analysis of *B* *. rapa* gene expression. In conclusion, genome-wide transcriptome analysis of Chinese cabbage requires the use of a *B* *. rapa* -specific microarray, instead of *Arabidopsis* chips.

### Analysis of microarray data

To identify genes with altered expression, including candidate GMS gene(s) and/or GMS-related genes in the Chinese cabbage, we carried out microarray analyses using the newly developed Br300K chip and RNAs from fertile and sterile buds (Table S3). Among 47,548 genes on the Br300K chip, 7,213 genes showed values of less than 500 in PI (probe intensity) from all tested floral bud samples. We ignored these genes in subsequent analyses. The remaining 40,335 genes were subjected to significance analysis of microarray (SAM) [47]. The false discovery cutoff was set at <5% and genes changing over 2-fold were selected. A total of 10,622 genes were differentially expressed; 4,774 genes were up-regulated over 2-fold in at least one of four fertile buds compared with sterile buds, while 5,848 genes were down-regulated (Table S3, S4). About 12–20% of the differentially expressed genes appeared to have no *Arabidopsis* counterparts, indicating that they might be present in *B* *. rapa* and/or other plants but not in *Arabidopsis* . Among the up-regulated genes in any stage of the fertile buds, 41% of them showed up-regulation in all stages, indicating that many genes may function in several developmental stages of pollen formation.

There were 11,390 clones that were classified as no hit found in the initial analysis with *Arabidopsis thaliana* annotation (Table S3). Among these, 293 clones were specifically expressed in fertile buds and only 28 clones in sterile buds (Table S5, S6). When these sequences were subjected to BLASTn, most of the F-specific clones showed similarity to *B. oleracea* (12), *B. napus* (15), and other plant clones (62). Seventy clones (56 fertile-specific and 14 sterile-specific) were matched only to *B* *. rapa* bacterial artificial chromosome (BAC) clone sequences, implying that they are specific to *B* *. rapa* and will be important for further research to discover novel GMS-related genes. In addition, several genes that were classified as unknown function but were specifically expressed in the fertile buds, such as Brapa_ESTC000796, Brapa_ESTC008117, and Brapa_ESTC049183, would be good candidates for GMS-associated genes.

To verify the general pattern of gene expression during pollen development, we selected genes showing the highest PI values in each of the floral buds, and carried out semi-quantitative RT-PCR (Figure S6, Table S7). As shown in Figure S6, most of the genes that showed the highest PI values in sterile buds were also expressed in fertile buds. In addition, genes showing the highest PI value in F1 and F2 buds were also expressed in sterile buds at very low levels. However, some genes from F2 buds were not expressed in sterile buds at all, indicating a possible involvement in male fertility. As expected, genes that had the highest PI value in F4 buds were specifically expressed in fertile buds. They started expression in the F2 buds and continued through to the F4 buds, the pollen maturation stage, indicating that, in GMS plants, expression of genes in late stages of pollen development may be inhibited.

### Genotype-specific expression of genes

In addition to being significantly different from SAM, genotype-specific genes were defined as genes that had PI values of over 1,000 in at least one bud type in a genotype, but less than 500 in all buds of other genotype, e.g., F-specific genes have a PI value of over 1,000 in any of the fertile buds (F1-F4 buds), but less than 500 in all three sterile buds (Table S8, S9). The total numbers of F- and S-specific genes were 1,413 and 199, respectively, implying that the expression of large numbers of genes which might be important for fertility was defective in GMS floral buds. Of the F-specific genes, 71% showed the highest expression in F4 buds, the pollen maturation stage, indicating that putative GMS genes affect the expression of many genes involved in the late stage of pollen development. Approximately 1%, 9%, and 17% of genes were highly expressed in F1 (before tetrad), F2 (at tetrad), and F3 (after tetrad) buds, respectively, indicating that 90% (1,272 genes) of the genes were highly expressed after the tetrad stage. By contrast, among the genes that were more highly expressed in the sterile buds, most (82%) were highly expressed at the tetrad stage.

**Figure 2:**
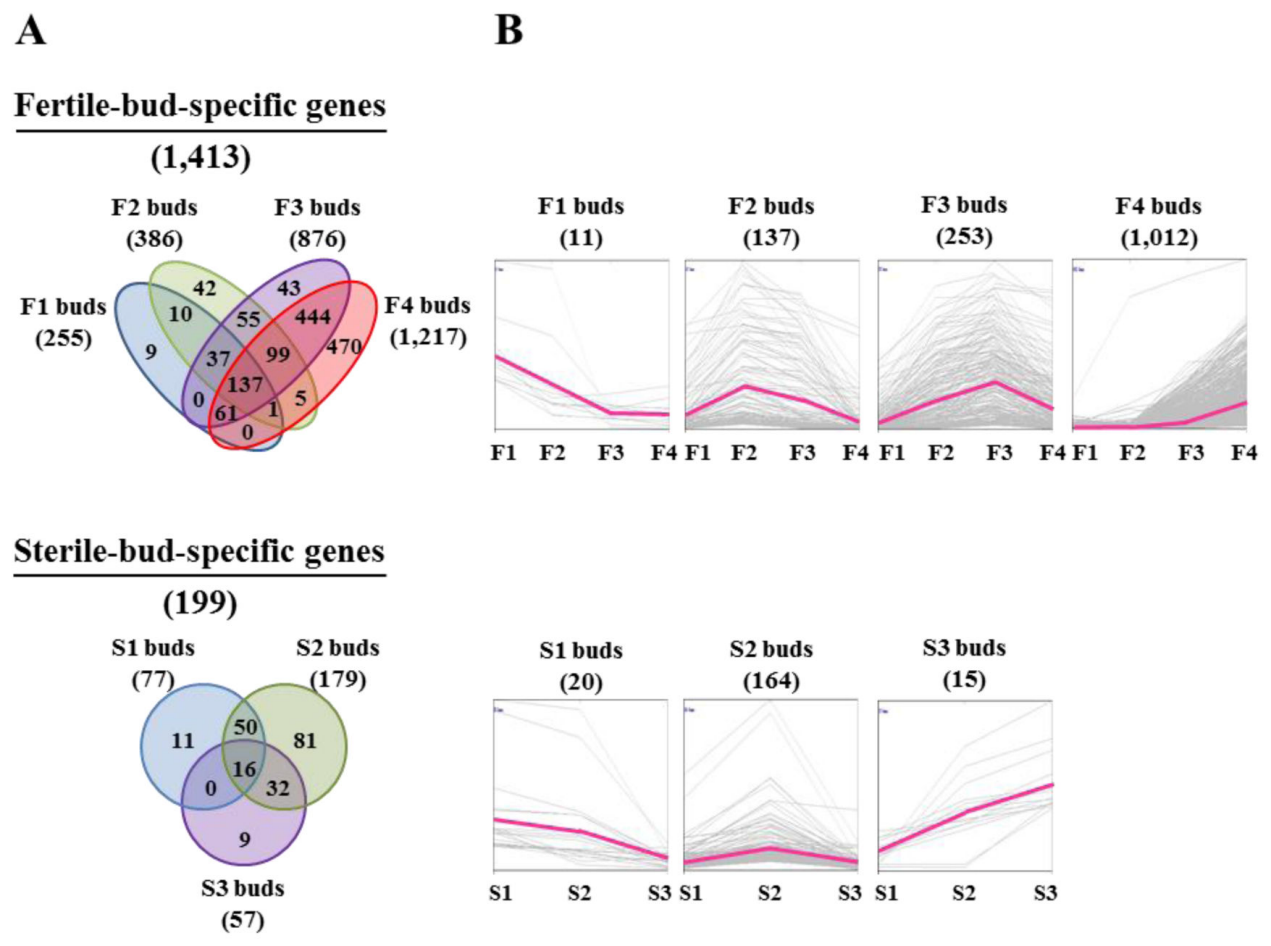
Distribution of genes expressed specifically according to genotype. A, Venn diagram of the distribution of genes expressed specifically according to genotype of Chinese cabbage. B, K-means clustering and graph format of the expression pattern of F- and S-specific genes. Pink colored lines indicate average PI values. The specific genes were classified into four F-specific gene clusters or three S-specific gene clusters by K-means clustering of MeV software (http://www.tm4.org/mev.html). The number in the brackets indicates the gene number of each cluster.

A Venn diagram and K-mean clustering of the genes listed in Tables S8 and S9 are shown in Figure 2. As shown in Figure 2A, genes with PI values over 1,000 in all four fertile buds and three sterile buds totaled 337 and 16, respectively. Genes showing the highest PI value in F1 buds were not expressed in F3 and F4 buds, suggesting that none of these were related to male gametogenesis in our GMS Chinese cabbage. These could be excluded from putative GMS genes. On the other hand, genes showing the highest PI values in F2 buds were expressed through the F3 bud stage (Figure 2B). Genes showing the highest PI values in F3 buds were also expressed in both F2 and F4 buds, indicating these genes could be related to GMS phenotypes. Genes showing the highest PI values in F4 buds commenced expression in F3 buds and dramatically increased their levels at the F4 bud stage. Genes showing the highest PI values in S1 buds were also expressed in S2 buds, whereas most genes showing the highest PI values in S2 buds were only expressed at that stage. Several genes showing the highest PI values in S3 buds were highly expressed in S2 buds as well. All of these data indicate that fertile or sterile bud-specific genes might function in a relatively broad range of pollen development. Otherwise, our samples include several stages of pollen development.

Genotype-specific genes were functionally grouped based on 'The Arabidopsis Information Resource; http://www.Arabidopsis.org/'. As shown in Table 2, most of the sterile bud-specific genes were highly expressed in S2 buds, the dominant categories of which were transferase activity, transcription factors, protein binding, and membrane metabolism. A high proportion of fertile bud-specific genes were associated with transporter activity, kinase activity, and lipid metabolic processes. In addition, F-specific genes were largely expressed in F4 buds.

**Table 2:** Functional categorization of F-and S-specific genes.

	**Fertile buds**						**Sterile buds**			
	**F1**	**F2**	**F3**	**F4**	**Total**		**S1**	**S2**	**S3**	**Total**
**Transporter activity**		6	11	84	**101**		3	5	1	**9**
**Kinase activity**	1	2	9	86	**98**		2	7		**9**
**Lipid metabolic process**	1	6	22	62	**91**			6	3	**9**
**Ion binding**		8	14	45	**67**		1	10		**11**
**Cell wall metabolism**		3	10	52	**65**			5		**5**
**Hydrolase activity**		2	8	52	**62**			6	2	**8**
**Membrane metabolism**		7	13	42	**62**			9	2	**11**
**Transferase activity**		9	11	38	**58**			13		**13**
**Catalytic activity**		6	8	41	**55**			6		**6**
**Protein binding**		13	12	28	**53**			10	1	**11**
**Carbohydrate metabolic process**		4	5	40	**49**			2	1	**3**
**Transcription factor**	3	2	6	33	**44**		1	11	1	**13**
**Response to stress**		4	7	31	**42**		2	9		**11**
**Signal transduction**			5	22	**27**			2		**2**
**Pollen tube growth**			1	24	**25**					**0**
**Proteolysis**			8	14	**22**			4		**4**
**Embryonic development**		1	4	16	**21**			1		**1**
**Pectate lyase activity**			2	17	**19**					**0**
**Oxidoreductase activity**		4	3	10	**17**			5		**5**
**Calcium signaling**				15	**15**			1		**1**
**Lyase activity**				13	**13**					**0**
**Pollen development**		1	2	8	**11**					**0**
**RNA processing**		1	3	7	**11**					**0**
**Protein myristoylation**		1		9	**10**			3	1	**4**
**Cell differentiation**		1		5	**6**					**0**
**Actin metabolism**				5	**5**					**0**
**electron carrier activity**				4	**4**			5		**5**
**Cytoskeleton organization**				3	**3**		1			**1**
**No clear classification**	4	37	54	114	**209**		8	27		**35**
**No_hit found**	1	17	34	94	**146**		2	17	3	**22**
**Total**	**10**	**135**	**252**	**1,014**	**1,411**		**20**	**164**	**15**	**199**

### Genes showing dramatically altered expression

The following categories were selected by both previous reports and highly altered gene groups found in this study: peroxidases (PODs), purple acid phosphatases (PAPs), multidrug and toxic compound extrusion (MATE) efflux family proteins, cytochrome P450 family proteins, lipid transfer protein (LTP) family, Cys-proteinase, kinases, transporters, and carbon supply-related genes.

Among 68 BrPOD genes, 14 (eight *Arabidopsis* counterparts) and eight (two *Arabidopsis* counterparts) genes were specifically expressed in sterile and fertile buds, respectively (Figure S7). These numbers, compared with their *Arabidopsis* counterparts, indicate that *BrPOD* genes are present in multiple copies in Chinese cabbage. Jiang et al. [48] reported that the expression level of reactive oxygen species (ROS)-scavenging genes was high during pollen development. However, major cell wall peroxidases reported by Bayer et al. [49] in *Arabidopsis* were highly expressed in both buds, implying that fertile bud-specific *PODs* found in this study might be novel genes expressed during pollen development in Chinese cabbage.

PAPs belong to a metallophosphoesterase superfamily and are characterized by their pink or purple color in solution [50]. Our microarray revealed that several *BrPAP* genes were highly and specifically expressed in either fertile or sterile buds of Chinese cabbage. Among 18 *BrPAPs* on the Br300K chip, three (*BrPAP3, 7*, and *8*) were specifically expressed in sterile buds, while another three (*BrPAP5, 6*, and *11*) were specifically expressed in fertile buds (Figure S7), suggesting that the latter three might play an important role in pollen development. In tobacco (*Nicotiana tabacum*), NtPAP12 is bound to the cell wall and enhances the activities of cellulose and callose synthases [51]. Due to sequence similarity among *PAP* genes in plants, we speculate that *BrPAP5, 6*, and *11* might have similar functions during pollen development to NtPAP12.

MATE family proteins are known to confer tolerance to toxins like aluminum in plants [52,53], and Chinese cabbage contains many MATE genes. Among 65 MATE efflux family protein genes on the Br300K chip, two and four genes (three *Arabidopsis* counterparts) were specifically expressed in sterile buds and fertile buds, respectively (Figure S7). The rest showed no significant difference between sterile and fertile buds. The role of MATE efflux proteins in pollen development is not clear, but their expression implies some sort of function of these genes related to the developmental process.

Numerous P450s have been known to be involved in the biosynthesis and metabolism of triterpenoids and steroids [54], the phenylpropanoid pathway [55], and lipid exine synthesis [8], all of which are required for normal pollen development. Among 311 cytochrome P450 (CYP) genes on the Br300K chip, 11 and 15 were specifically expressed in sterile and fertile buds, respectively (Figure S8). In particular, seven fertile bud-specific genes (which were similar to seven *Arabidopsis* counterparts) (*BrCYP71B2, BrCYP86C2, BrCYP86C3, BrCYP86C4, BrCYP705A24, BrCYP707A3*, and *BrCYP735A1*) were first reported as pollen development-related P450s in this study. The *CYP98A8* gene, mentioned by Matsuno et al. [55], was not F-specific, but its expression levels were 14–287-fold increased (in an allelic-specific manner) in the fertile buds. However, the upstream gene of *CYP98A8, BrSHT* (spermidine hydroxycinnamoyl transferase, AT2G19070), was specifically and highly expressed in the fertile buds, indicating possible involvement in pollen fertility.

The transport of lipid molecules from the tapetum to the microspore surface has been considered to be an essential process for the pollen wall formation. LTPs are basic extracellular small (9 kDa) proteins present in high amounts (as much as 4% of the total soluble proteins) in higher plants [56] and are involved in the fertilization process, such as pollen tube growth, pollen allergens, and pollen tube adhesion [57,58]. Among 116 LTP family genes on the Br300K microarray, five (three *Arabidopsis* counterparts) and 18 (nine *Arabidopsis* counterparts and five *Brassica* -specific genes) were specifically expressed in sterile and fertile buds, respectively (Figure S9). A previous report found that LTP types 1 and 2 (At3g51590 and At1g66850) were significantly reduced in the *Arabidopsis* *ams* mutant [59]. The fertile bud-specific expression of *B* *. rapa* genes homologous to these LTPs might imply the importance of their function in pollen development after meiosis. *BrATA7* in particular, which has 70% identity to the *A. thaliana* anther-specific gene 7 (AT4G28395) [60] at the amino acid sequence level, would be another candidate GMS gene.

Since several Cys proteases and their inhibitors are thought to be involved in PCD in tapetum [59,61–64], it can be assumed that Cys-proteinases are important in pollen development in Chinese cabbage. Among 50 Chinese cabbage Cys-proteinase genes, 12 genes (corresponding to three *Arabidopsis* genes; AT1G06260, AT2G31980, and At4G36880) were highly and specifically expressed in fertile buds (Figure S9). These fertile-bud-specific genes might be related to pollen development in Chinese cabbage. Some of these have not been mentioned in other male sterile plants, implying the presence of PCD regulatory pathways that differ from those of *Arabidopsis* . The swollen tapetum layer might also be caused by the inhibition of PCD [65], resulting from defective *AtMYB103/80, MS1*, and *AMS* [20,37–39]. On the other hand, the swollen tapetum layer observed in Figure 1 might be influenced only by transcription factor *AMS* (Table 3) and various proteinase genes.

**Table 3:** Summary of known gene expression levels in *Arabidopsis* and Chinese cabbage used in this study.

	** *Arabidopsis* **			** *Arabidopsis* microarray data**							** *Brassica* *rapa* ssp. *pekinensis* **				
**Classification**	**Gene Name**	**Locus**	**Description**	**WT/*ems1*^1^**	**WT/*spl*^1^**	**WT/*tdf1*^2^**	**WT/*ms1*^3^**	**WT/*ams*** ^4^		**WT/*bri*** ^5^	**F1/S1**	**F2/S2**	**F3/S3**	**F4/S3**	** *B* *. rapa* Seq. Id**
								**Meiosis**	**Mitosis I**						
**Stamen formation**	*AP2*	AT4G36920	APETALA 2	-1.2	-4.9	-2.2	.	.	.		1.0	1.0	1.1	1.1	Brapa_ESTC034160, 13840, 07967
	*LFY*	AT5G61850	LEAFY	-1.5	-2.9	.	1.5	.	.		-1.2	1.0	-1.3	-1.3	Brapa_ESTC036995
	*AG*	AT4G18960	AGAMOUS	.	.	.	.	.	.		-1.1	-1.2	1.1	-1.1	Brapa_ESTC044174, 8198,18123, 08506
**Microsporangium differentiation**	*NZZ/SPL*	AT4G27330	SPOROCYTELESS	1.6	13.8	.	.	.	.	1.9	1.0	2.0	15.9	4.1	Brapa_ESTC020996
**(Early anther development)**	*EMS1*	AT5G07280	EMS1 (EXCESS MICROSPOROCYTES1); kinase	7.9	2.1	.	.	.	.		-1.1	2.1	2.6	-1.8	Brapa_ESTC029822
	*BAM1*	AT5G65700	Big apical meristem 1; protein serine/threonine kinase	.	.	.	.	.	.		1.0	1.1	-1.5	-2.0	Brapa_ESTC012414, 06935
	*BAM2*	AT3G49670	Big apical meristem 2	.	.	.	.	.	.		1.0	1.0	-1.4	-1.3	Brapa_ESTC043430
	*SERK1*	AT1G71830	SOMATIC EMBRYOGENESIS RECEPTOR-LIKE KINASE 1	.	.	.	.	.	.		1.0	1.1	-1.1	-1.2	Brapa_ESTC033477, 27479, 14825, 40476
	*ATMKK3*	AT5G40440	MITOGEN-ACTIVATED KINASE KINASE 3	.	.	.	.	.	.		1.0	1.2	1.0	-1.1	Brapa_ESTC024122, 19250,20760
	*ATMPK6*	AT2G43790	MAP KINASE 6	.	.	.	.	.	.		1.0	-1.1	-1.2	1.0	Brapa_ESTC014784, 08095
	*ERL1*	AT5G62230	ERECTA-LIKE 1; kinase	-1.5	-2.1	.	.	.	.		-1.1	-1.1	-1.3	-1.2	Brapa_ESTC025460
	*ERL2*	AT5G07180	ERECTA-LIKE 2; kinase	-1.5	-2.6	.	.	.	.		-1.1	1.0	-1.3	-1.6	Brapa_ESTC002620
	*ROXY1*	AT3G02000	ROXY1; thiol-disulfide exchange intermediate	-1.6	-2.6	-2.0	.	.	.		-1.2	1.1	-1.4	-2.2	Brapa_ESTC042441
	*ROXY2*	AT5G14070	Glutaredoxin family protein	4.4	29.0	.	.	.	.		-1.8	-1.6	-2.3	-1.6	Brapa_ESTC045661
**Early tepetum development**	*MS5*	AT4G20900	MALE-STERILE 5	3.4	3.1	.	.	.	.		-2.1	-2.1	-1.8	-1.3	Brapa_ESTC043424
	*MS5*-like	AT1G04770	Male sterility MS5 family protein	.	.	.	.	-1.5	1.8		-1.1	-1.1	1.6	1.1	Brapa_ESTC020157, 15922, 04737, 12635, 16503, 07564
	*MS5*, putative	AT3G51280	Male sterility MS5, putative	.	.	.	-1.7	.	.		-1.1	-1.1	1.0	-2.0	Brapa_ESTC043512
	*MS5*-like	AT5G44330	Male sterility MS5 family protein	6.2	6.0	.	.	.	.		1.5	-1.7	-2.9	-2.7	Brapa_ESTC038820
	*MS5*-like	AT5G48850	Male sterility MS5 family protein	.	.	.	.	.	.		-1.1	1.1	2.5	-1.1	Brapa_ESTC031499, 16710, 22399, 13812, 00358
	*MYB4*	AT4G38620	MYB4	.	.	.	.	.	.		-1.5	-1.6	-1.1	-1.2	Brapa_ESTC018007
	*AtMYB35*	AT3G28470	AtMYB35(TDF: Tapetal Development and Function 1)	40.6	61.5	.	-3.6	.	.		1.1	1.1	-1.6	-1.7	Brapa_ESTC037115
		AT3G13220	ABC transporter family protein	52.2	56.7	2.2	.	5.9	0.0		1.2	1.7	-1.1	-2.0	Brapa_ESTC033269, Brapa_ESTC000274
	*P450*	AT1G69500	Oxygen binding (P450)	117.5	129.0	9.2	-2.5	11.6	0.0		2.1	1.3	-1.7	-17.8	Brapa_ESTC040440, Brapa_ESTC000961
	*MYB103/MYB80*	AT5G56110	AtMYB103/AtMYB80	2.2	2.5	.	.	.	.	19.8	1.5	1.4	-2.1	-2.8	Brapa_ESTC046330
	*bHLH89*	AT1G06170	Basic helix-loop-helix (bHLH) family protein 89	38.7	79.4	.	.	2.6	1.9		1.3	6.2	155.3	36.0	Brapa_ESTC015754, Brapa_ESTC020728
**Tapetum development**	*AtMYB65*	AT3G11440	AtMYB65	1.3	4.7	.	.	.	.		1.0	1.8	2.4	1.6	Brapa_ESTC036883
	*MS1*	AT5G22260	MALE STERILITY 1	.	.	.	.	.	.	17.3	4.4	3.8	-1.2	-2.0	Brapa_ESTC027135
	*AMS*	AT2G16910	ABORTED MICROSPORES	31.8	28.8	3.7	.	.	.	4.8	1.3	1.7	17.2	6.3	Brapa_ESTC025857, 11209, 10964
	*AtMYB99*	AT5G62320	AtMYB99	2.5	2.9	.	2.8	.	.		63.0	26.5	2.6	-1.5	Brapa_ESTC028843
	*ATA1*	AT3G42960	*Arabidopsis* TAPETUM 1; oxidoreductase	61.3	7.7	3.0	.	8.2	0.0	13.0	1.9	1.2	1.2	-19.1	Brapa_ESTC015748, 08703
	*ATA7*	AT4G28395	*Arabidopsis thaliana* anther 7	8.3	11.9	31.1	10.7	6.0	7.6		243.2	74.4	218,8	9.5	Brapa_ESTC011088, 44558
	*ATA20*	AT3G15400	*Arabidopsis thaliana* anther 20	21.6	57.2	4.8	.	12.3	20.7		4.3	3.8	46.4	14.0	Brapa_ESTC050089, 49943
	*ATGPAT1/GPAT1*	AT1G06520	GLYCEROL-3-PHOSPHATE ACYLTRANSFERASE 1	26.8	47.8	.	.	.	.		1.0	1.0	3.4	2.9	Brapa_ESTC017885, 17205
	*MS2*	AT3G11980	MALE STERILITY 2; fatty acylreductase	42.8	50.9	29.8	.	17.5	11.4	11.4	2.4	1.4	1.4	-18.6	Brapa_ESTC048175, 01042,10283, 08439
	*MEE48 (A6)*	AT4G14080	Maternal effect embryo arrest 48	82.9	204.5	53.9	-3.4	12.0	0.0		1.6	1.2	1.1	-14.5	Brapa_ESTC008631, 08374, 17985,08727, 08365, 28775, 01024
	*A9*	AT5G07230	Protease inhibitor/seed storage/lipid transfer protein family protein (**A9**)	50.8	221.1	40.1	.	13.7	1.5		1.8	3.6	2.4	-7.6	Brapa_ESTC001846, 00106
	*ATLP-3*	AT1G75030	*Arabidopsis* thaumatin-like protein 3	15.9	56.2	2.3	.	5.9	0.0		2.3	1.2	1.8	1.6	Brapa_ESTC034925, 34897, 02604, 18634, 34926
	*QRT3*	AT4G20050	QRT3 (QUARTET 3)	34.4	37.2	5.4	.	6.2	6.0		3.1	3.1	13.0	8.1	Brapa_ESTC025970, 08657
	*AtMYB32*	AT4G34990	AtMYB32	5.1	2.5	.	.	.	.		1.0	-1.1	-1.5	1.4	Brapa_ESTC020465, 10344, 30500
**Pollen wall development**	*ANAC025*	AT1G61110	*Arabidopsis* NAC domain containing protein 25	2.8	3.4	7.7	11.4	4.2	10.3		11.6	80.0	20.2	11.3	Brapa_ESTC010704, 20348
	*LTP12*	AT3G51590	LIPID TRANSFER PROTEIN 12	11.4	31.0	28.9	51.9	12.8	7.8	2.7	112.3	66.7	139.4	19.1	Brapa_ESTC047756, 01668, 00931, 28789, 26972, 00864, 01664, 49901
	*Beta-1,3-glucanase*	AT3G23770	Glycosyl hydrolase family 17 protein e)	28.5	25.0	5.6	5.7	.	.		3.3	1.6	-2.9	-7.1	Brapa_ESTC008581, 43265, 08350, 08384
	*PAB5*	AT1G71770	POLY(A)-BINDING PROTEIN	7.5	13.9	.	.	0.0	-1.9		-1.3	2.8	18.6	23.3	Brapa_ESTC033470, 47603, 07874, 20721, 28732, 17836
	*FLP1/WAX2*	AT5G57800	FLP1/WAX2; catalytic	.	.	.	.	.	.		1.1	1.5	1.6	1.1	Brapa_ESTC034677, 07038, 34675, 10368, 34678, 09965
	*LAP3*	AT3G59530	Strictosidine synthase family protein	2.6	10.9	.	.	.	.		1.3	1.2	11.1	4.9	Brapa_ESTC011139, 27142, 43884
	*DEX1*	AT3G09090	DEFECTIVE IN EXINE FORMATION 1	.	.	.	.	.	.		1.0	1.2	-1.3	-1.3	Brapa_ESTC016224, 07010, 18363
	*DEX2*	AT1G01280	CYP703A2 (cytochrome P450, family 703, subfamily A, polypeptide 2)	47.9	43.6	14.6	-3.8	8.6	0.0		1.9	1.7	-3.0	-4.0	Brapa_ESTC020422, 11063, 32856, 18250
	*ATMYB103*	AT1G63910	ATMYB103	.	.	.	.	.	.		1.1	1.0	2.3	3.3	Brapa_ESTC031325
** *B* *. rapa* MS genes**	*BcMF2; PGA4*	AT1G02790	BcMF2; PGA4 (POLYGALACTURONASE 4)	16.8	9.1	44.6	24.3	0.0	1.3		12.8	6.6	58.1	125.4	Brapa_ESTC008069, 19365, 09311, 07709, 28587, 09221, 39243, 08239
	*BcMF7*	AT1G04670	Unknown protein	.	.	3.7	.	.	.		-1.8	3.8	64.2	88.9	Brapa_ESTC028237, 15704
	*BcMF12*	AT1G14530	TOM THREE HOMOLOG	.	.	.	.	.	.		1.6	2.3	2.2	2.4	Brapa_ESTC035970
	*BcMF9*	AT3G07820	Polygalacturonase 3 (PGA3) / pectinase	13.6	4.5	30.0	.	0.0	11.3		9.0	4.7	37.2	128.5	Brapa_ESTC009239
	*BcMF6*	AT5G48140	Polygalacturonase, putative / pectinase, putative	.	.	20.6	.	0.0	0.0		8.2	6.9	42.0	93.2	Brapa_ESTC007655
**Putative GMS gene**	*EXL6*	AT1G75930	Extracellular lipase 6	2.2	2.7	15.8	26.2	0.0	28.5		92.8	113.9	258.6	170.4	Brapa_ESTC010981
	*ATA27*	AT1G75940	Catalytic/ cation binding / hydrolase (beta-glucosidase)	5.9	12.2	7.0	51.9	14.7	23.2		53.8	115.5	163.9	47.6	Brapa_ESTC004210
		AT1G73860	ATP binding/ microtubule motor	.	.	.	.	.	.		1.1	2.6	17.3	32.9	Brapa_ESTC037859
	*ASK2*	AT3G61160	Shaggy-related protein kinase beta / ASK-beta	.	.	11.8	.	.	.		1.7	2.8	22.8	21.4	Brapa_ESTC005304
**AMS-dependent genes**	*ABC transporter*	AT3G13220	ABC transporter family protein	.	.	.	.	2.6	2		1.5	1.8	.	.	Brapa_ESTC000274
	*CHS*	AT4G00040	Chalcone and stilbene synthase family protein					-2.0	1.3		5.4	13.9	105.4	12.6	Brapa_ESTC000529, 17929, 20778

Genes were selected on the basis of previous reports of *Arabidopsis* mutants and Chinese cabbage mutants affecting anther or pollen development. All values are expressed in terms of the ratio of wild type to mutant, so that positive values indicate depression of gene expression in mutants. Dots represent either no difference or no expression. Data for Chinese cabbage were obtained by recalculation, i.e., mean values were used if there were multiple genes.

1 1954 genes that are differentially expressed in *spl* and *ems1*mutants (Wijeratne et al., 2007)

2 1327 genes changing *tdf1* mutant (Zhu et al., 2008)

3 966 genes changing in *ms1* mutant (Yang et al., 2007)

4 Genes changing in *ams* mutants (Xu et al., 2010)

5 Genes changing in *bri* mutants (5Ye et al., 2010).

Extracellular invertase genes (also known as cell wall invertases or beta-fructofuranosidases) were expressed specifically in anther and they supplied carbohydrate to the developing microspores [66]. Repression of or interference with extracellular invertase caused male sterility, while complementation restored fertility [66]. *Arabidopsis* contains six cell wall invertases (*AtcwINV1–AtcwINV6*) (At3g13790, At3g52600, At1g55120, At2g36190, At3g13784, and At5g11920) [67]. Among these, *AtcwINV2, 4*, and *5* were expressed in flower and/or seeds, while *AtcwINV1, AtcwINV3*, and *AtcwINV6* were expressed in all tissues [67]. In our microarray data, the counterparts of *AtcwINV1* and *AtcwINV3* were expressed in all floral buds, while that of *AtcwINV6* was not expressed in floral buds (data not shown). However, the counterpart of *AtcwINV2* was highly expressed in F4 buds, indicating that its function may be important in pollen development at the late stage (Figure S9).

**Figure 3:**
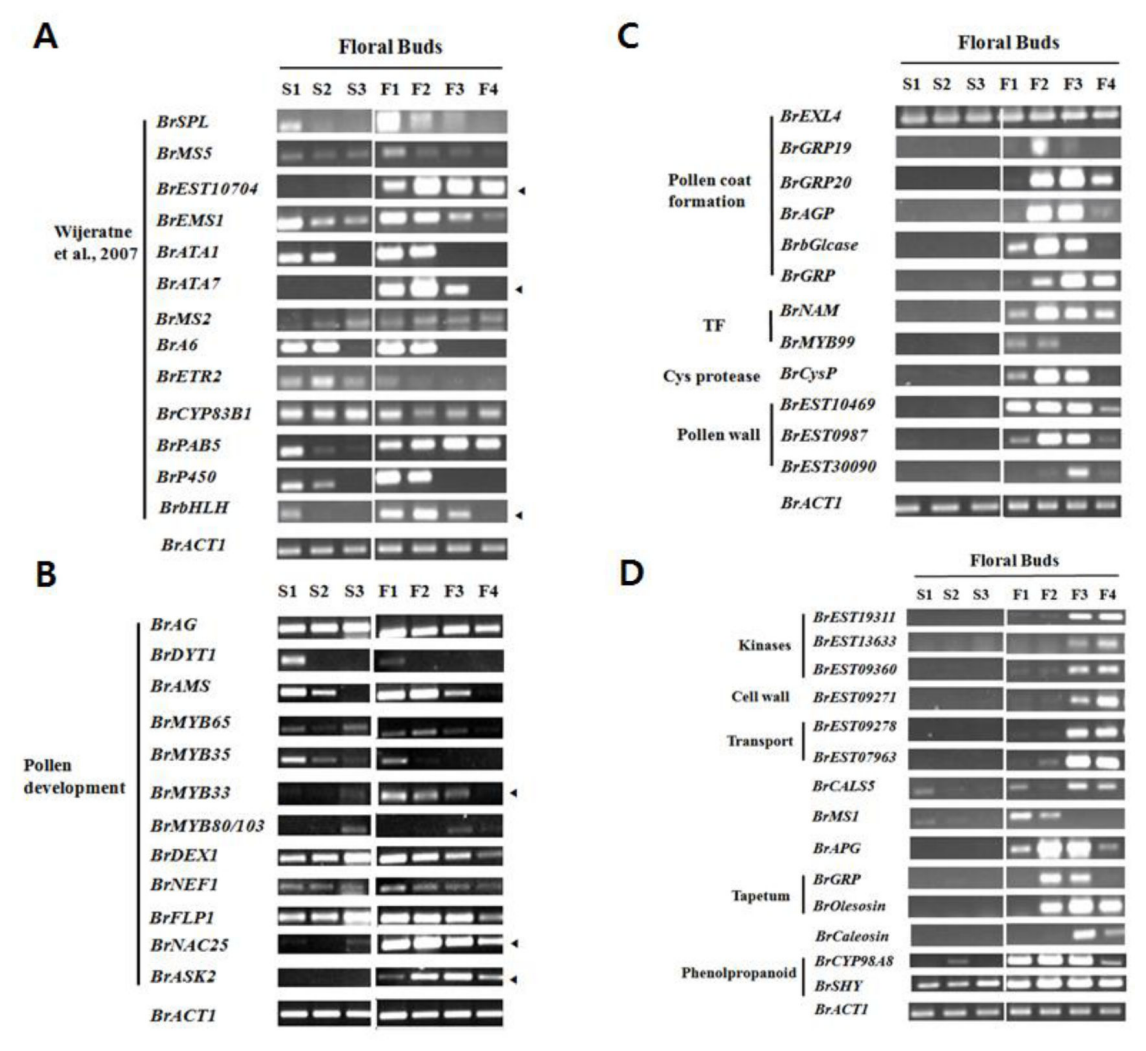
Expression of genes previously identified in male sterile mutants of *Arabidopsis* and other *Brassica* species. A, Major genes mentioned by Wijeratne et al., 2007. B, Other pollen development-associated genes identified in *Arabidopsis* . C and D, Late pollen development-associated genes identified in *Arabidopsis* and *Brassica* species. Arrows indicate putative GMS-associated genes.

Kinases and phosphatases are major regulatory components that control various pathways. This fact naturally leads to the presumption of involvement of these gene products in pollen development. Particularly, receptor-like protein kinases regulated male sterility from the early stages [64,68,69] to the late pollen developmental stage [70]. Among 1,226 protein kinase genes on the 300K chip, 63 of them, including those mentioned in *Ms-cd1 B. oleracea* by Kang et al. [23] were differentially expressed (Table S10). All receptor-like kinase genes were expressed in fertile buds, showing the highest expression level in F4 buds. In particular, receptor-like kinase genes (counterparts of AT3G21910, AT3G21920, 3G21930, AT3G21990, AT3G22040, AT3G29040, and AT3G58310) were highly expressed and up-regulated in the fertile buds, implying a critical role in pollen development. ASK1 ( *Arabidopsis* SKP1-like 1) is a component of Skp1-Cullin-F1-box-protein (SCF) complexes involved in protein degradation by the 26S proteasome. It also plays a role in male meiosis [71,72]. Knockout of the *ask1* gene in *Arabidopsis* caused male sterility [71]. In this study, no difference in *BrAsk1* expression was observed between sterile and fertile buds (Table S1). However, *BrASK2* appears to be essential for male fertility (Figure 3), supporting the hypothesis that either our GMS occurs after meiosis of the male gametophyte, or that different regulatory mechanisms for fertility operate between the two species. In other words, BrASK2 appears to have taken over BrASK1 function in *B* *. rapa* .

Kang et al. [23] found that many transporter genes were down-regulated in male sterile *B. oleracea*. Counterparts of those mentioned by Kang et al. [23] were highly up-regulated in the fertile buds of Chinese cabbage (Table S11), indicating possible involvement in pollen fertility. In addition, three sugar transporter genes (monosaccharide transporter, *BrSTP9*; sugar transporter family protein, AT4G04760; and putative sugar transporter, AT4G02050) and two amino acid transporter genes (aromatic and neutral transporter 1, *BrANT1*; and Lys/His transporter 7, *BrLHT7*) were also expressed specifically in fertile buds. Cation/hydrogen exchangers 8, 13, 14, 19, 25, and 27 (*BrCHX 8, BrCHX 13, BrCHX 14, BrCHX19, BrCHX25*, and *BrCHS27*) were found to be highly and specifically expressed in fertile buds. Responsive-to-antagonist1 (*BrRAN1*), K^+^ ATPase1 (*BrKAT1*), vacuolar H^+^ ATPase (*BrVHA-E2*), AAA-type ATPase family protein genes, and P-glycoprotein 10, 11, and 12 (*BrPGP10-12*) were also highly and specifically expressed in fertile buds. One transporter gene (AT1G31885 counterpart) was expressed specifically in F2 and F3 buds. All of these data imply that pollen development requires sugars, amino acids, and ions in Chinese cabbage, similar to *B. oleracea*.

In addition, it was reported that *Arabidopsis* magnesium transporter family member, *AtMGT9*, which functions as a low-affinity Mg^2+^ transporter, has a crucial role in male gametophyte development and male fertility [24]. In our microarray data, three alleles belong to this transporter family. One (Brapa_ESTC020685) showed no difference in its expression between sterile and fertile buds, but two (Brapa_ESTC020255 and Brapa_ESTC046558) were up-regulated in fertile buds, specifically, F2 and F3 buds. Particularly, Brapa_ESTC046558 seems to display fertile-specific expression, implying that it might be involved in male fertility.

### Pollen wall and coat formation genes

After microspore release from the tetrad, formation of the pollen wall and the pollen coat are major events controlled by the tapetum layer and microspores. Based on cytological study (Figure 1), a change in the expression of numerous genes involved in pollen wall and coat formation in GMS floral buds (Tables 4-5) seemed to be the result of defects in an early event in male gametophyte development. These genes might participate in the fertilization process.

**Table 4:** Expression of genes associated with cell wall formation and modification.

**Locus**	**Proposed function**	**F1/S1**	**F2/S2**	**F3/S3**	**F4/S3**	**Chip ID**
At1g10770	Invertase/pectin methylesterase inhibitor family protein	7.1	2.9	23.8	98.4	Brapa_ESTC009277, 07659, 35873, 27289, 19381
At1g23350	Invertase/pectin methylesterase inhibitor family protein	1.0	1.6	7.4	41.9	Brapa_ESTC009310, 30079
At1g48020	Invertase/pectin methylesterase inhibitor family protein	5.4	2.6	47.1	239.7	Brapa_ESTC000154, 38232, 15678
At1g54620	Invertase/pectin methylesterase inhibitor family protein	1.1	1.1	51.7	115.5	Brapa_ESTC046143, 46162
At1g60760	Invertase/pectin methylesterase inhibitor family protein	1.1	-1.3	21.2	72.0	Brapa_ESTC019401, 17851
At2g01610	Invertase/pectin methylesterase inhibitor family protein	-1.2	1.0	1.1	14.6	Brapa_ESTC033170
At2g47050	Invertase/pectin methylesterase inhibitor family protein	8.2	4.0	26.2	84.0	Brapa_ESTC001202, 07925, 42142, 09328
At2g47670	Invertase/pectin methylesterase inhibitor family protein	-1.4	-1.4	2.0	35.4	Brapa_ESTC042188
At3g17220	Invertase/pectin methylesterase inhibitor family protein	1.7	1.0	17.4	136.1	Brapa_ESTC017267
At3g36659	Invertase/pectin methylesterase inhibitor family protein	5.1	8.2	13.2	105.0	Brapa_ESTC028827
At3g62180	Invertase/pectin methylesterase inhibitor family protein	2.2	2.1	24.5	63.1	Brapa_ESTC017808, 09312, 02602
At4g02250	Invertase/pectin methylesterase inhibitor family protein	3.3	2.2	10.2	53.6	Brapa_ESTC045243, 09356, 17166
At5g46930	Invertase/pectin methylesterase inhibitor family protein	1.0	-1.9	-1.1	17.6	Brapa_ESTC046139
At5g50030	Invertase/pectin methylesterase inhibitor family protein	3.4	1.8	6.3	124.9	Brapa_ESTC026039, 09218
At1g69940	ATPPME1; Pectinesterase	5.1	3.0	29.3	61.1	Brapa_ESTC029837, 08127, 27087, 17215
At2g47040	VGD1 (VANGUARD1); Pectinesterase	12.7	7.0	48.9	114.0	Brapa_ESTC027331, 47221, 07956, 09301, 17681
At3g62170	VGDH2 (VANGUARD 1 HOMOLOG 2); Pectinesterase	7.6	5.6	43.3	106.5	Brapa_ESTC011048, 10367, 38300, 00162, 17840, 17194, 11233
At4g24640	APPB1; Pectinesterase inhibitor	-1.2	-1.5	-1.1	31.4	Brapa_ESTC033815
At2g26450	Pectinesterase family protein	5.3	2.9	7.6	60.3	Brapa_ESTC019329, 09281
At2g47030	Pectinesterase family protein	17.0	12.9	62.4	141.2	Brapa_ESTC001194
At3g05610	Pectinesterase family protein	29.2	108.7	272.1	207.9	Brapa_ESTC008173, 09355, 37604
At3g06830	Pectinesterase family protein	1.1	-1.1	2.2	37.3	Brapa_ESTC026016, 27294, 25419, 42619
At3g17060	Pectinesterase family protein	5.3	2.7	9.8	80.5	Brapa_ESTC009333, 19399, 09255
At4g33230	Pectinesterase family protein	-1.3	-1.3	1.8	29.4	Brapa_ESTC044869
At5g07410	Pectinesterase family protein	1.4	1.1	50.9	169.5	Brapa_ESTC017088, 17602
At5g07420	Pectinesterase family protein	4.8	4.0	28.6	47.1	Brapa_ESTC009260
At5g07430	Pectinesterase family protein	8.4	2.9	13.1	109.7	Brapa_ESTC009228, 09331, 50417, 50418
At5g49180	Pectinesterase family protein	5.0	2.8	11.1	60.0	Brapa_ESTC009229, 26027, 17017, 19289
At1g75940	ATA27 (*Arabidopsis thaliana* anther 27)	70.2	332.3	296.8	50.3	Brapa_ESTC004210, 07739
At3g62710	Glycosyl hydrolase family 3 protein	2.9	1.4	4.0	31.8	Brapa_ESTC009374, 09346
At5g16580	Glycosyl hydrolase family 1 protein	3.9	12.7	7.6	1.9	Brapa_ESTC034720
At5g54570	Glycosyl hydrolase family 1 protein	1.3	1.4	20.9	9.5	Brapa_ESTC017471
At1g02310	Glycosyl hydrolase family protein 5	-1.5	-4.6	-5.8	3.1	Brapa_ESTC005598
At3g43860	Glycosyl hydrolase family 9 protein	6.8	4.3	10.5	86.5	Brapa_ESTC009354, 09371
At4g23560	Glycosyl hydrolase family 9 protein	1.0	1.2	1.6	26.2	Brapa_ESTC044430
At5g64790	Glycosyl hydrolase family 17 protein	2.2	1.0	13.3	54.3	Brapa_ESTC027328, 19366, 46577, 09248
At2g05790	Glycosyl hydrolase family 17 protein	38.7	134.4	503.1	124.2	Brapa_ESTC007538, 06532
At5g17200	Glycoside hydrolase family 28 protein	25.1	6.1	-2.3	-2.4	Brapa_ESTC045761, 17864
At1g65590	Glycosyl hydrolase family 20 protein	3.0	9.5	1.7	2.4	Brapa_ESTC002982, 50349, 35437, 35436
At4g35010	BGAL11 (beta-galactosidase 11)	4.6	3.0	28.0	83.7	Brapa_ESTC009323, 26008, 19413, 27299, 09381, 28620, 07643
At2g16730	BGAL13 (beta-galactosidase 13)	2.6	2.0	8.3	73.2	Brapa_ESTC009266, 07699, 19310
At2g23900	Glycoside hydrolase family 28 protein	3.2	3.1	35.0	136.3	Brapa_ESTC027329, 11332
At3g07820	Polygalacturonase 3 (PGA3) / pectinase	9.0	4.7	37.2	128.5	Brapa_ESTC009239
At1g02790	PGA4 (Polygalacturonase 4); Polygalacturonase	12.8	6.5	58.1	125.4	Brapa_ESTC009221, 08239, 07709, 09311, 08069, 19365, 28587, 39243
At1g02790	PGA4 (POLYGALACTURONASE 4)	18.1	6.3	227.2	1179.7	Brapa_ESTC003812
EU181170	*Brassica* *rapa* pollen-specific polygalacturonase	10.4	10.2	40.8	60.2	Brapa_ESTC047193
At3g07840	Polygalacturonase, putative / pectinase, putative	6.9	4.7	52.4	100.4	Brapa_ESTC025822, 26049, 08394, 07902, 18295, 13597
At5g48140	Polygalacturonase, putative / pectinase, putative	4.7	4.5	64.7	165.8	Brapa_ESTC007655, 28667
At3g07830	Polygalacturonase, putative / pectinase, putative	10.0	9.6	177.5	318.9	Brapa_ESTC000552
At3g07850	Exopolygalacturonase	1.3	2.0	104.3	263.5	Brapa_ESTC008094
At3g14040	Exopolygalacturonase	1.0	1.6	45.6	114.1	Brapa_ESTC010586, 42779, 28006
At5g15110	Pectate lyase family protein	3.1	1.8	6.7	65.8	Brapa_ESTC027367, 27350, 09271, 30679, 10996
At3g01270	Pectate lyase family protein	5.6	2.8	19.7	80.1	Brapa_ESTC046917, 42401, 08189, 26034, 09342
At2g02720	Pectate lyase family protein	2.6	1.5	7.1	60.7	Brapa_ESTC042247, 26015, 09231, 09351, 28567
At3g52600	CWINV2 (CELL WALL INVERTASE 2)	1.6	2.0	2.1	17.4	Brapa_ESTC034099, 09236, 27284, 09304, 05384
At1g14420	AT59 ( *Arabidopsis* homolog of tomato LAT59)	5.6	3.4	13.6	60.4	Brapa_ESTC009294, 19322, 39628, 09379, 27276, 19330
At5g14380	AGP6 (ARABINOGALACTAN PROTEINS 6)	1.5	2.0	83.2	315.9	Brapa_ESTC001855, 45636
At3g01700	AGP11 (ARABINOGALACTAN PROTEIN 11)	7.1	3.4	22.0	62.7	Brapa_ESTC001198, 10226, 42427
At3g12660	FLA14 (Fasciclin-like arabinogalactan protein 14 precursor)	1.1	-1.5	84.3	147.7	Brapa_ESTC011072
At3g57690	AGP23 (ARABINOGALACTAN-PROTEIN 23)	6.3	7.4	23.2	46.2	Brapa_ESTC028155, 28022, 00826, 47834, 35077
At3g20865	AGP40 (ARABINOGALACTAN-PROTEIN 40)	3.5	2.5	15.4	52.9	Brapa_ESTC030338, 27969
At5g24105	AGP41	3.6	2.1	7.2	33.2	Brapa_ESTC028029, 48514, 28985, 48513, 34435
At2g41905	Similar to AGP23 (ARABINOGALACTAN-PROTEIN 23)	8.2	6.3	26.4	48.9	Brapa_ESTC028027, 48519, 03480, 48520

All values are expressed in terms of the ratio of wild type to mutant, so that positive values indicate depression of gene expression in mutants. Dots represent either no difference or no expression. Data for Chinese cabbage were obtained by recalculation, i.e., mean values are used if there are multiple genes.

#### 1) Pollen cell wall formation genes

Since the formation and modification of the pollen cell wall is also important for normal pollen development, we analyzed microarray data related to two categories: cell wall modification-related genes and cell wall arabinogalactan proteins (AGPs). A large number of genes involved in pollen cell wall formation and modification were specifically expressed in fertile buds.

Cell wall modification-related genes include six families: methyltransferase, pectate lyase, pectinesterase family, polygalacturonase, glycosyl hydrolase, and fructosidase genes. Five hundred and twenty-three Chinese cabbage clones contain such genes. Among these, 158 were highly expressed in fertile buds, including all genes mentioned by Kang et al. [23]. However, the degree of up-regulation was much higher in Chinese cabbage (up to 1,004-fold) than *B. oleracea* (31-fold) (Table 4). Fourteen invertase/pectin methylesterase inhibitor family protein genes, 14 pectinesterase genes, 11 glycosyl hydrolase family protein genes, 8 polygalacturonase genes, and 5 pectate lyase family protein genes were highly and specifically expressed in fertile buds. These results are similar to those of the *B. oleracea* experiment, but the level of expression was more dramatic and many novel genes might be induced in Chinese cabbage. *BrPGA4* (polygalacturonase 4) and *BcMF2* (At1G02790 homolog) have many alleles in Chinese cabbage, the expression of which showed two patterns: one group was highly expressed in F3 and F4 buds, but expression of the others began in F1 buds and continued to F4 buds. Interestingly, among the invertase/pectin methylesterase inhibitor family protein genes, counterparts of AT1G23350 (Brapa_ESTC009310, Brapa_ESTC030079, and Brapa_ESTC019649) and AT1G60760 (Brapa_ESTC019401, Brapa_ESTC019401, and Brapa_ESTC017851) showed both up- and down-regulation in fertile buds (Table S8, S9), suggesting the existence of allelic-specific expression patterns.

To release microspores from the early PMC stage, several specialized PMC wall layers must be generated and degraded [35]. *Ms-cd1 B. oleracea*, similar to our GMS, exhibited degradation of the primary PMC wall and delayed degradation of callose surrounding the tetrads, thereby arresting microspore release [23]. In our microarray data, two important enzymes for the degradation of esterified and unesterified pectin, pectin methylesterase (PME) and polygalacturonase (PG), were differentially expressed, whereas callose degradation genes were not, indicating little difference in the mechanism underlying male sterility. One putative PG gene, *Brassica campestris* Male Fertility 9 (*BcMF9*), conferred male fertility by acting as a coordinator in the late stages of tapetum degeneration, and subsequently in the regulation of wall material secretion and, in turn, exine formation [8]. In our microarray, its homolog also showed altered expression, with high levels in F3 and F4 buds, suggesting an important role in GMS.

Alpha 1-acid glycoproteins (AGPs) connect the plasma membrane to the cell wall [73]. They are a family of extensively glycosylated hydroxyproline-rich glycoproteins located on the cell surface. They are required for stamen and pollen development and function [73,74]. Therefore, it was expected that Chinese cabbage AGPs might be also involved in male fertility. Similar to *Arabidopsis* data, *BrAGP6*, *BrAGP11, BrAGP14, BrAGP23, BrAGP40, BrAGP41, and BrAGP23* were highly expressed in fertile buds, particularly F3 and F4 buds. However, expression of the remaining 19 *BrAGPs* (*BrAGP1-4, BrAGP8-10, BrAGP12-16, BrAGP18-22*, and *BrAGP26* and *27*) showed no difference between fertile and sterile buds (Table 4). These data indicate that at least six *AGPs* could be associated with pollen development in Chinese cabbage.

#### 2) Pollen coat-related genes

The pollen coat of the family Brassicaceae, including *A. thaliana, B. napus*, *B. oleracea*, and *B* *. rapa* , consists of lipids and proteins that facilitate adhesion to insect vectors and mediate pollen-stigma interactions during pollination and fertilization processes [75,76]. Lipases and oleosins (largely oleo-pollenins) are major protein components (over 90%) of the pollen coat [76,77], while protein kinases and pectin esterase are minor components [76].

Pollen coat lipases are largely composed of GDSL lipases and extracellular lipases (EXLs) [77,78]. Among 95 clones encoding GDSL lipase genes from Chinese cabbage, three genes (corresponding to two *Arabidopsis* genes) and 13 genes (corresponding to nine *Arabidopsis* genes) were specifically expressed in sterile and fertile buds, respectively (Table 5). The remaining genes were either not expressed or constitutively expressed in both floral buds. On the other hand, 58 genes belonging to extracellular lipases and other lipases were found in the Br300K microarray. Among these, 3 and 51 genes were specifically expressed in sterile and fertile buds, respectively (Table 5). *BrEXL4, BrEXL6*, and the putative family II *EXLs* were highly expressed in the fertile buds. Interesting findings included a very highly up-regulated gene, encoding a beta-ketoacyl-CoA synthase family protein, which catalyzes wax synthesis, in fertile buds (F1, F2, and F3 buds). Another interesting finding was that the acyl-activating enzyme 11 (*AAE11*) gene was highly expressed only in S3 and F4 buds.

**Table 5:** Expression of genes associated with pollen coats and pollen itself.

**Classification**	**Locus**	**Proposed function**	**F1/S1**	**F2/S2**	**F3/S3**	**F4/S3**	**Chip Id**
**Lipases**	At1g53990	GLIP3 (GDSL-motif lipase 3)	-8.3	-26.1	-4.0	-22.8	Brapa_ESTC009454
	At1g33811	GDSL-motif lipase/hydrolase family protein	-2.1	-2.2	-3.1	-2.8	Brapa_ESTC019974,09492
	At1g08310	Esterase/lipase/thioesterase family protein	-1.7	-4.6	-5.5	-4.4	Brapa_ESTC021270
	At4g01950	ATGPAT3/GPAT3 (GLYCEROL-3-PHOSPHATE ACYLTRANSFERASE 3)	-1.8	-5.5	-2.0	-3.8	Brapa_ESTC038354
	At1g06990	GDSL-motif lipase/hydrolase family protein	33.1	169.2	143.0	37.7	Brapa_ESTC030587
	At2g03980	GDSL-motif lipase/hydrolase family protein	3.7	14.3	31.3	61.7	Brapa_ESTC025896,26051,19325,09337,030427
	At2g19050	GDSL-motif lipase/hydrolase family protein	-1.1	3.9	1.0	3.8	Brapa_ESTC019358
	At2g19060	GDSL-motif lipase/hydrolase family protein	1.4	4.1	-1.1	4.3	Brapa_ESTC019277
	At4g30140	GDSL-motif lipase/hydrolase family protein	-1.6	-1.3	-6.3	2.9	Brapa_ESTC016082
	At5g42160	GDSL-motif lipase/hydrolase protein-related	1.2	1.1	55.8	158.2	Brapa_ESTC007977
	At5g55050	GDSL-motif lipase/hydrolase family protein	1.6	-1.5	-1.1	2.7	Brapa_ESTC046261
	At4g16230	GDSL-motif lipase/hydrolase family protein	6.6	4.6	-1.8	-2.9	Brapa_ESTC044100
	At4g18970	GDSL-motif lipase/hydrolase family protein	1.6	1.2	1.6	1.0	Brapa_ESTC005372
	At5g55050	GDSL-motif lipase/hydrolase family protein	1.0	-1.1	-1.6	1.4	Brapa_ESTC046261,09754,02525,46258,25660,14932
	At4g24230	ACBP3 (ACYL-COA-BINDING DOMAIN 3)	1.6	3.2	1.0	-1.4	Brapa_ESTC036645
	At1g06250	Lipase class 3 family protein	9.8	26.6	33.8	8.3	Brapa_ESTC001825,38095,17220
	At1g20120	Family II extracellular lipase, putative	6.5	90.5	174.8	67.1	Brapa_ESTC003556,08527
	At1g20130	Family II extracellular lipase, putative	57.9	254.7	596.6	184.9	Brapa_ESTC010869,11093,00842,17410,00950,07743,07731
	At1g52570	PLDALPHA2 (Phospholipase D alpha 2)	10.2	34.7	169.1	40.6	Brapa_ESTC008744
	At1g75930	EXL6 (Extracellular lipase 6); acyltransferase/ carboxylic ester hydrolase/ lipase	84.4	108.0	299.7	186.7	Brapa_ESTC010981,03525
	At2g31100	Lipase, putative	2.5	27.1	44.1	20.0	Brapa_ESTC021123,17575,25890
	At3g26820	Esterase/lipase/thioesterase family protein	25.5	125.2	119.2	26.5	Brapa_ESTC018145
	At1g20132	Hydrolase, acting on ester bonds / Lipase	124.9	191.1	217.1	1.9	Brapa_ESTC047743
	At1g75910	EXL4 (Extracellular lipase 4); acyltransferase/ carboxylic ester hydrolase/ lipase	3.4	84.8	155.7	66.2	Brapa_ESTC008149
	At5g42170	Family II extracellular lipase, putative	-1.1	1.0	102.4	497.8	Brapa_ESTC007775
	At2g45610	Unknown protein	-1.2	-1.3	12.8	55.9	Brapa_ESTC035916
	At3g19310	Phospholipase C	2.7	1.6	5.0	50.1	Brapa_ESTC007768,27332
	At4g11030	Long-chain-fatty-acid--CoA ligase, putative / long-chain acyl-CoA synthetase, putative	1.0	3.3	20.8	42.5	Brapa_ESTC017722,30260
	At4g34510	KCS2 (3-ketoacyl-CoA synthase 2); acyltransferase	1.5	1.0	15.0	177.0	Brapa_ESTC017633
	At5g20410	MGD2 (monogalactosyldiacylglycerol synthase 2)	1.9	3.3	6.2	20.2	Brapa_ESTC027309
	At2g24320	Unknown protein	1.1	-1.1	1.9	20.4	Brapa_ESTC020321,22840
	At2g39420	Esterase/lipase/thioesterase family protein	1.4	1.4	2.0	10.5	Brapa_ESTC026359,29494
	At2g40116	Phosphoinositide-specific phospholipase C family protein	-1.0	-1.4	1.0	13.8	Brapa_ESTC020217
	At3g43550	Carboxylic ester hydrolase/ lipase	-1.1	-1.1	1.0	57.0	Brapa_ESTC011330
	At4g29460	Phospholipase A2 gamma	5.6	1.7	8.8	95.6	Brapa_ESTC009383
	At5g14180	Lipase family protein	1.0	1.4	2.8	13.3	Brapa_ESTC045651
	At2g42010	PLDBETA1 (Phospholipase D beta 1)	1.0	-2.0	1.2	13.3	Brapa_ESTC027306
	At2g20900	Diacylglycerol kinase, putative	1.4	1.1	4.0	5.3	Brapa_ESTC027113
	At3g11430	ATGPAT5/GPAT5 (GLYCEROL-3-PHOSPHATE ACYLTRANSFERASE 5)	-1.0	-3.3	-1.7	14.7	Brapa_ESTC036915,27319,26953,16969
	At1g08510	FATB (FATTY ACYL-ACP THIOESTERASES B)	3.4	1.5	1.3	2.8	Brapa_ESTC005825
	At3g52160	Beta-ketoacyl-CoA synthase family protein	13.3	55.7	32.9	1.0	Brapa_ESTC010783
**Oleosin/GRP**	At1g55990	Glycine-rich protein	-2.4	-12.7	-8.5	-9.8	Brapa_ESTC044904
	X96409	B.oleracea mRNA for pollen coat oleosin	74.8	736.2	1519.1	1309.0	Brapa_ESTC003529
	AY028608	B. oleracea transcription factor-like protein/pollen coat oleosin-glycine rich protein	21.0	96.8	98.1	33.8	Brapa_ESTC049223
	AY028608	B. napus STA 41-9; B. transcription factor-like protein; B. oleracea pollen coat oleosin	83.3	658.0	1183.6	1145.6	Brapa_ESTC000519
	AY028608	B. napus STA 41-9; B. transcription factor-like protein; B. oleracea pollen coat oleosin	28.3	167.6	172.6	191.0	Brapa_ESTC028636
	At5g07550	Pollen coat oleosin-glycine rich protein [Brassica oleracea]/GRP19	92.2	185.0	259.7	101.9	Brapa_ESTC002624
	At5g07550.2	GRP19 (Glycine rich protein 19)	7.5	96.4	120.2	10.3	Brapa_ESTC048968,48967,29655
	At5g07600	Oleosin / glycine-rich protein	5.3	153.3	350.2	233.4	Brapa_ESTC008160,01657,29653,29652
	At3g01570	Glycine-rich protein / Oleosin	3.8	3.2	-2.4	-2.0	Brapa_ESTC012713
	At5g07530	GRP17 (Glycine rich protein 17)	4.4	45.9	27.3	9.5	Brapa_ESTC008272
	At5g07550.1	GRP19 (Glycine rich protein 19)	45.5	463.4	888.4	34.4	Brapa_ESTC011474
	At5g61610	Glycine-rich protein / Oleosin	2.1	20.1	44.8	7.7	Brapa_ESTC018054
	At5g07560	GRP20 (Glycine rich protein 20); nutrient reservoir	1.7	188.1	371.0	268.2	Brapa_ESTC028013,29656,28646
	At2g25890	Glycine-rich protein / Oleosin	1.7	6.0	94.2	157.7	Brapa_ESTC027006
	At1g23240	Caleosin-related family protein	1.1	1.2	205.7	252.5	Brapa_ESTC008102
	Y08986	B.napus gene encoding oleosin-like protein (TF)	1.4	12.4	91.3	61.4	Brapa_ESTC047095
	Y08986	B.napus gene encoding oleosin-like protein (TF)	-1.5	20.3	181.8	97.7	Brapa_ESTC029651
	Y08986	B.napus gene encoding oleosin-like protein (TF)	9.4	218.4	180.5	8.3	Brapa_ESTC029654
	X82020	B.nappus mRNA for oleosin (pol3)	2.0	97.2	310.1	209.4	Brapa_ESTC000518
	X82020	B.nappus mRNA for oleosin (pol3)	4.2	281.6	975.5	575.2	Brapa_ESTC003555
	X82020	B.nappus mRNA for oleosin (pol3)	2.5	342.7	719.2	564.3	Brapa_ESTC003686
	X67142	B. napus C98 mRNA (oleosin)	35.5	355.4	1080.3	502.2	Brapa_ESTC003622
	NtF	Brassica napus tapetal oleosin-like (BnOlnB;4) gene	-2.7	15.9	111.5	96.7	Brapa_ESTC000792
	EF079958	Brassica rapa oleosin-like protein mRNA	1.6	51.4	83.3	52.8	Brapa_ESTC029658
	EF079958	Brassica rapa oleosin-like protein mRNA	1.8	111.7	114.1	85.7	Brapa_ESTC007884
	EF079958	*B* *. rapa* * oleosin-like protein mRNA*	4.8	388.6	774.4	641.9	Brapa_ESTC017377
	AY028608	Brassica oleracea transcription factor-like protein (T2I1_290) gene	56.1	782.9	2194.9	1253.0	Brapa_ESTC003611
	AY028608	B. oleracea transcription factor-like protein (GRP1, 2, 3, 4, 5)	16.2	87.3	407.1	123.2	Brapa_ESTC046974
	At3g18570	Glycine-rich protein / Oleosin	19.7	342.6	872.6	526.0	Brapa_ESTC043156,13099,22398,33810
	U77666	*B* *. rapa* pollen coat protein homolog (BAN103)	2.7	4.5	123.3	328.1	Brapa_ESTC049819,48528,49820,48527
	At3g21920	Pollen coat receptor kinase, putative /receptor-like kinase-related	58.9	157.8	145.6	61.1	Brapa_ESTC028841
**Pollen**	At1g24520	BCP1 (Brassica campestris pollen protein 1)	6.8	3.2	10.4	53.5	Brapa_ESTC028066,09216
	At3g13400	Putative pollen-specific protein mRNA	0.9	1.6	7.4	25.1	Brapa_ESTC047835
	At5g39400	Pollen specific phosphatase, putative / phosphatase and tensin, putative (PTEN1)	1.5	3.7	4.4	54.1	Brapa_ESTC045901,10448
	At3g03430	Polcalcin, putative / calcium-binding pollen allergen, putative	1.0	1.2	32.8	86.2	Brapa_ESTC042503,06474
	At5g17480	Polcalcin, putative / calcium-binding pollen allergen, putative	2.1	4.6	135.0	223.4	Brapa_ESTC003820,45786
	At3g13400	Putative pollen-specific protein	0.9	1.6	7.4	25.1	Brapa_ESTC047835
	At4g18596	Pollen Ole e 1 allergen and extensin family protein	7.5	3.9	17.8	70.9	Brapa_ESTC044263,07645,29019,09224,26025
	At5g45880	Pollen Ole e 1 allergen and extensin family protein	11.3	4.7	32.8	110.6	Brapa_ESTC009367,09376,26064
	At1g29140	Pollen Ole e 1 allergen and extensin family protein	2.6	2.0	31.4	86.8	Brapa_ESTC040131,25887,20687,08222,07664
	At3g26110	BCP1 (Brassica campestris pollen protein 1)	13.7	3.0	25.9	449.3	Brapa_ESTC001598
	At2g25600	SPIK (SHAKER POLLEN INWARD K+ CHANNEL)	2.2	3.3	6.5	51.8	Brapa_ESTC041235

All values are expressed in terms of the ratio of wild type to mutant, so that positive values indicate depression of gene expression in mutants. Dots represent either no difference or no expression. Data for Chinese cabbage were obtained by recalculation, i.e., mean values are used if there are multiple genes.

Oleo-pollenins (oleosin-like proteins) made up 50–80% of total pollen coat proteins by mass, whereas oleosins and calosins are minor components of the pollen coat [76]. The oleo-pollenins include many from the glycine-rich protein (GRP) family [75,79]. In our microarray data, one *BrGRP* (AT1G55990 homolog) gene was expressed specifically in sterile buds. However, 35 genes were specifically and highly expressed in fertile buds (Table 5), which included *Arabidopsis* counterparts, *B. napus* homologs, *B. oleracea* homologs, and *B* *. rapa* genes. Only one of these is the calosin-related family proteins.

Pectin esterases and protein kinases are less-abundant proteins in the pollen coats that facilitate the penetration of the emerging pollen tube into the stigmatic surface and that participate in signaling processes, respectively [76]. In our microarray data, one pollen coat receptor-like kinase (AT3G21920 homolog) and one Chinese cabbage pollen coat protein homolog (*BAN103*) (U77666) showed fertile bud-specific expression (Table 5). Particularly, the receptor-like protein kinase might play a role in an entire stage of normal pollen development.

In addition to the above proteins, our microarray data revealed that genes encoding five pollen-specific proteins, one phosphatase, two polcalcins, three pollen Ole e 1 allergens, and one channel were specifically and highly expressed in fertile buds. These data indicate that in addition to cell wall and pollen coat proteins, many pollen components are required for male sterility or male gametophyte development (Table 5). Although many genes essential for the formation of both pollen wall and coat were suppressed in GMS, the pollen maturation and anther dehiscence would be expected to be normal since the expression of genes essential for late stage pollen development, such as *PM-ANT1*, *ER-ANT1*, and mitochondrial ATP/ADP carriers *AAC1* and *AAC2* [80], was high in all S1-3 and F1-4 floral buds.

### Expression analysis of transcription factors

**Figure 4:**
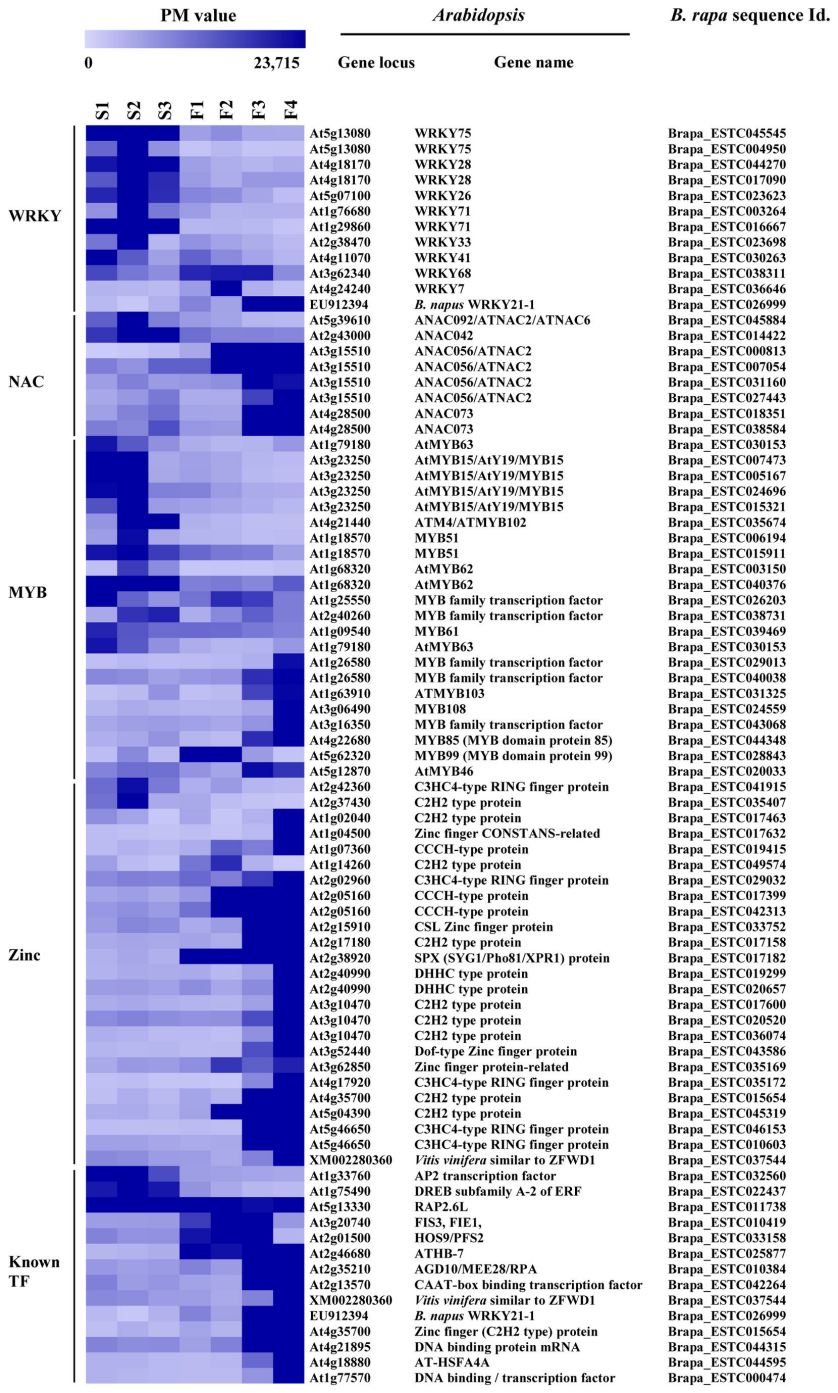
Hierarchical cluster display of the transcription factors in Chinese cabbage. The color scale bar shown above the cluster indicates the maximum and minimum brightness values that represent the PI value.

Transcription factors can regulate a number of genes associated with a specific trait, so their effects will be more powerful than those of structural genes. We analyzed several major transcription factors showing altered expression in GMS Chinese cabbage (Figure 4). Among 56 BrWRKY transcription factor genes, seven genes (*BrWRKY26*, *BrWRKY28, BrWRKY33, BrWRKY41*, two *BrWRKY71*, and *BrWRKY75*) were expressed specifically in sterile buds, whereas three genes (*BrWRKY7, BrWRKY21-1*, and *BrWRKY 68*) were expressed specifically in fertile buds. In particular, *BrWRKY21-1* (homologous to *B. napus WRKY21-1* [81]) was highly expressed in F3 and F4 buds, implying a possible involvement in pollen development and/or pollen fertility.

NAC [for NAM (no apical meristem), ATAF1, 2, CUC2 (cup-shaped cotyledon 2)] transcription factors are one of the largest plant TF families. They share an N-terminal NAC domain. Since NAC transcription factors have been found to be key regulators of stress perception and developmental programmes [82], examining their expression profiles could provide insight into their involvement in pollen development. A total of 66 NAC transcription factors were analyzed in this microarray. Among them, two (*BrNAC42* and *BrNAC92*) were expressed in sterile buds, while another two (*BrNAC56* and *BrNAC73*) were expressed in fertile buds. Two *BrNAC56* (Brapa_ESTC000813 and Brapa_ESTC007054) homologs of *NARS2/NAC2*, which regulates embryogenesis in *Arabidopsis* [83], were expressed from F2 to F4 floral buds, whereas two novel BrNAC73 (Brapa_ESTC01835 and Brapa_ESTC038584) genes were expressed in F3 and F4 floral buds, indicating possible involvement in pollen development. The remaining 47 genes were constitutively expressed in both types of buds, but 15 genes were not expressed in the tested tissues.

Among 279 *BrMYB* transcription factor genes, 14 (9 *Arabidopsis* genes) and 8 (7 *Arabidopsis* genes) were specifically expressed in sterile and fertile buds, respectively. *BrMYB46, BrMYB85, BrMYB99, BrMYB103* (*MYB80* or *MS188*)*, BrMYB108*, and two *MYB* genes appeared to be fertile bud-specific. Interestingly, most fertile bud-specific *MYB* genes were highly expressed in F4 buds, whereas *BrMYB99* was highly and specifically expressed in F1 and F2 buds. This *BrMYB99* will be a putative candidate for control of the early stage of Chinese cabbage GMS, while others will be putative candidates for pollen fertility.

Among 1,542 zinc finger family protein genes deposited on the Br300K chip, 2 and 23 genes were specifically expressed in sterile and fertile buds, respectively. Two sterile bud-specific genes are C3H4-type RING finger and C2H2 type (*BrZAT11*) genes, while fertile bud-specific genes are comprised of C2H2-, C3H3-, CCH-, DHHC-, and Dof-type protein genes. Among these, C2H2-type family protein genes are remarkably highly expressed in F3- and F4- buds.

Analysis of known transcription factors revealed two (AT1G33770 and AT1G75490 homologs) and 11 (*FIS3, HOS9/PF2, ATHB-7, AGD10/MEER28/RPA*, *MSG2/IAA19, ZFWD1, At-HSF4A*, AT4G35700, AT4G21895, and AT1G77570 homologs) genes that were specifically expressed in sterile and fertile buds, respectively. Most of these are associated with dehydration stress and ovule development. In contrast to our data, none of these genes has been reported to be related to male fertility, implying that more functions than those related to pollen development should be elucidated.

### Prediction of gene function through analysis of expression profiling during floral bud development

**Figure 5:**
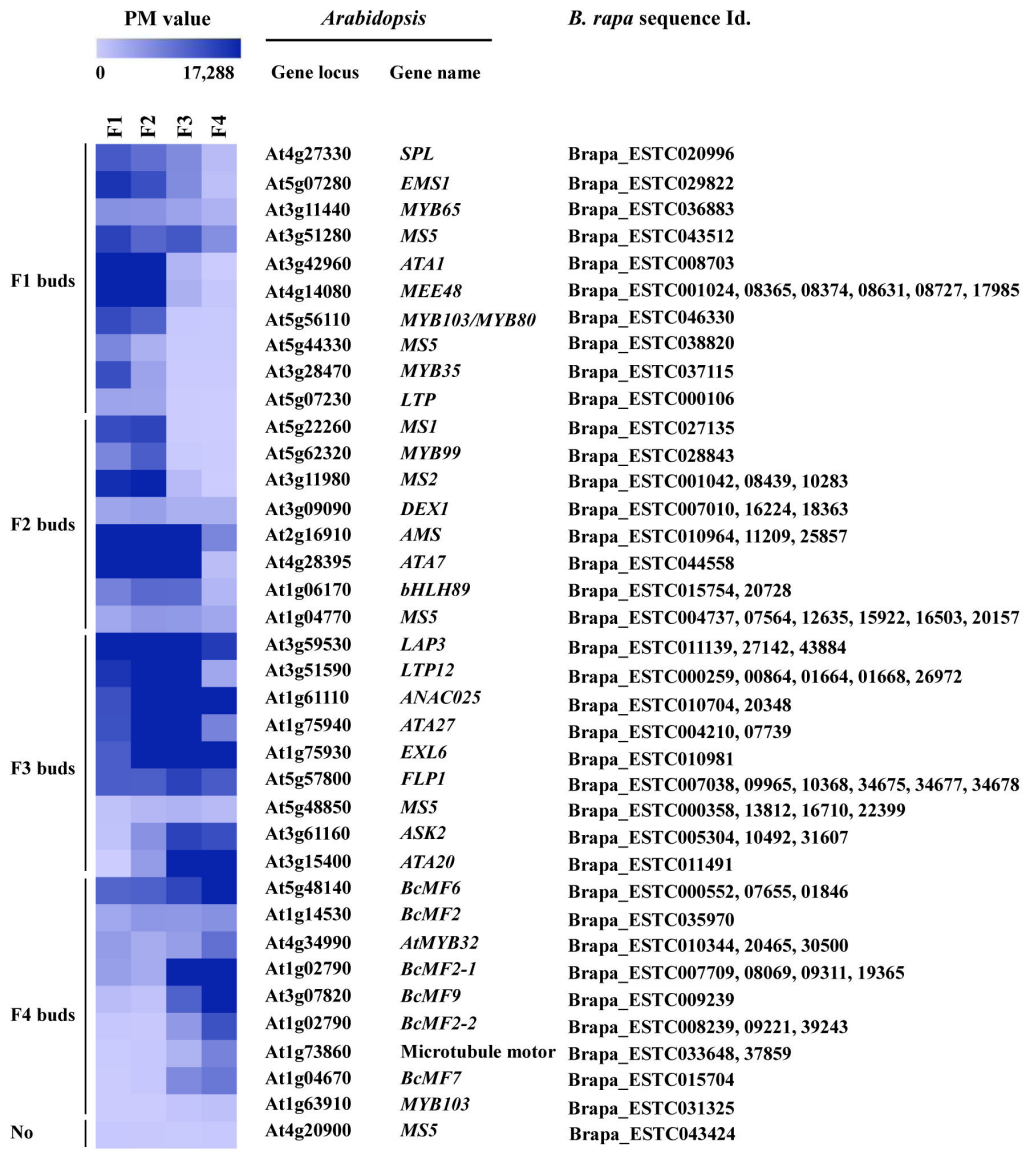
Hierarchical cluster display of pollen development-associated genes in Chinese cabbage. The color scale bar shown above the cluster indicates the maximum and minimum brightness values that represent the PI value.

Analysis of gene expression levels (expressed as PI values) during floral bud development provides an opportunity to identify sequentially operating genes and to predict the function of previously known genes in other plant systems. As shown in Figure 5, the somewhat similar regulatory pathway underlying *Arabidopsis* pollen development might also exist in Chinese cabbage. The expression of *BrNZZ/SPL* and *BrEXS/EMS1* began in F1 buds and continued through to the pollen maturation stage F4. Interestingly, *BrMYB103/MYB80*, one of the *BrMS5s*, *BrMYB35*, LTP family protein gene, *BrMS1*, and *BrMYB99* were expressed only in F1 and F2 floral buds, not in F3 and F4 buds. In addition, the transcript levels for *BrMS2* and *BrATA1* were high in F1 and F2 buds, but not detectable in F4 buds. On the other hand, the transcripts for *BrATA20*, microtubule motor gene, *BcMF7*, and *BrMYB103* were not detectable in F1 buds. According to Figure 5, the chronological working order of floral bud developmental genes in Chinese cabbage should be different from that in *Arabidopsis* . BrMYB35 and BrMYB103/80 definitely worked upstream of BrMS1 and BrMYB99. BrMS1, BrMS2, and BrAMS might function at similar stages of pollen development.

As *Arabidopsis* contains multiple copies of the male sterility 5 (MS5) gene [84], the Br300K microarray includes five *BrMS5* genes: homologs of AT1G04770, AT3G512890, AT4G20900, AT5G44330, and AT5G48850 (*ATSDI1*; sulfur deficiency-induced 1). Unlike the *Arabidopsis* AT4G20900 gene, which when mutated led to male sterility [84], the transcript level of its homolog could not be detected in any of the seven floral buds, suggesting that it is not related to pollen development in Chinese cabbage. Instead, AT5G44330 and AT3G51280 might be functional, but they were also expressed in all sterile buds, indicating that they might not be major determinants in GMS even though they are required for pollen development. The counterpart of AT5G48850, the expression of which was highest in F3 buds, was also expressed in all seven floral buds, indicating that *MS5* genes do not play a critical role in Chinese cabbage GMS. All *BcMF* genes showed the highest expression levels in F4 buds. However, some of them were expressed in all floral buds, but others were expressed only in F3 and F4 buds. *Arabidopsis* *BES1* (BRI1-EMS-SUPPRESSOR1), an important transcription factor for brassinosteroid signaling, is considered to be a master gene that controls many transcription factors essential for anther and pollen development as well as MS1-downstream genes [40]. However, four homologs (Brapa_ESTC001714, Brapa_ESTC013323, Brapa_ESTC021551, and Brapa_ESTC039699) of *Arabidopsis* *BES1* were highly expressed in all seven floral buds (Table S3), indicating that the mechanism underlying GMS is different from that of *Arabidopsis* .

Tetrad formation defectives of *Arabidopsis* *, AtPC1* (*Parallel Spindle 1*) (At1G34355), and *JASON* (At1G0660) [85] were expressed in both sterile and fertile floral buds in our GMS (Table S3), indicating that the meiosis II or tetrad formation process would be normal or other genes may be involved in it.

### Comparison of *B. rapa* GMS with *Arabidopsis* MS genes

Genes regulating anther and pollen development in *Arabidopsis* have been well established by genetic and molecular biological studies. To unravel whether *B* *. rapa* GMS is also controlled by homologs of *Arabidopsis* genes, the alteration of expression of those genes was compared with previous results (Table 3). Genes associated with stamen formation, microsporangium differentiation (except *NZZ/SPL* and *EXS/EMS1*), and early tapetum development (except *bHLH89*) were not down-regulated in *B* *. rapa* GMS buds, indicating putative GMS gene(s) might be functioning downstream of these groups of genes. However, alteration of *NZZ/SPL* and *EXS/EMS1* expression in GMS might imply the presence of different pathways in the two plants. Other early genes associated with anther development in *Arabidopsis* , such as *MS5* [84], *MYB33*, and *MYB65* [86] showed no change in their expression in Chinese cabbage. The rice *UNDEVELOPED TAPETUM1* gene and its putative *Arabidopsis thaliana* ortholog *DYSFUNCTIONAL TAPETUM1* (*DYT1*), encoding basic helix-loop-helix (bHLH) transcription factor, are crucial for tapetal differentiation and the formation of microspores [35,87]. The *B* *. rapa* ortholog of *Arabidopsis* *DYT1* was absent in our microarray, but *BrDYT1* (*Bra013519* [The *Brassica* *rapa* Genome Sequencing Project Consortium, 2011] [88]), which was 86% identical to the *Arabidopsis* ortholog, was not expressed in any floral buds (data not shown). Instead, another bHLH transcription factor, *BrbHLH89*, might replace DYT1 function in Chinese cabbage (Table 3). Among major genes essential for post-meiotic tapetal function that are controlled by DYT1 [28,35,36], *MS1* and *AMS* appear to be related to GMS, but *MYB35* and *MYB103/80* do not (Figure 5, Table 3).

Most genes related to later pollen development were down-regulated in GMS floral buds, but some genes, such as *ATA1, MS2, ATLP-3, AtMYB32*, and *DEX2*, were not. In addition, expression of several genes associated with pollen wall development, such as *FLP1* and *DEX2*, was high in all seven buds. These data imply that exine formation genes are expressed in GMS buds, even in the aborted pollen grains.

AMS, a basic helix-loop-helix (bHLH) transcription factor, plays a role in completion of meiosis [38], and regulates 13 genes involved in anther development, including lipid transport and metabolism [59]. *BrAMS* showed altered expression, especially in F3 and F4 buds. The *Brassica* genome may contain two (or three) copies of *AMS* (*Bra002004* and *Bra030041*) (http://brassicadb.org) and both showed similar patterns of expression, but *Bra030041* (Brapa_ESTC011209 and Brapa_ESTC010964) changed to a greater degree. *B* *. rapa* GMS showed somewhat similar phenotypes to the *Arabidopsis* *ams* mutant, such as reduced filament length, swollen tapetum layer, and no pollen production. However, BrGMS revealed the failure of tetrad formation and release, indicating that additional genes are involved in this. *BrAMS* was expressed in both S1 and S2, but not in S3. In addition, *BrAMS* expression was high in F3 and F4 buds. This indicates that the *BrAMS* gene itself might be normal, but that signaling that controls *BrAMS* transcription could be disturbed in GMS buds. An ortholog of another *bHLH* gene, *bHLH89* (At1G06170), revealed a more dramatic change in GMS, indicating a more important role than *BrAMS* in GMS. Interestingly, both *bHLH* genes were highly expressed in S1, S2, F1, and F2 buds, but completely suppressed in S3 while keeping relatively high levels in F3 and F4 buds. This result indicates that upstream component(s) might play a major role in GMS. Another interesting finding was that the expression of chalcone synthase (*CHS*) was AMS-dependent, but that the expression of ABC transporter *WBC27* (AT3G13220) was not AMS-dependent in GMS. Since both genes were direct targets of AMS and essential for pollen fertility [59] in *Arabidopsis* , our data indicate somewhat different pollen development processes between the two plants.

### qRT-PCR confirmation of microarray analysis

To confirm our microarray data, we selected several genes that had been previously identified in *Arabidopsis* and other *Brassica* species. Transcript levels of these genes were examined by semi-quantitative RT-PCR (Figure 3). Some genes identified in *Arabidopsis* *spl* and *ems* mutants [14] were expressed in both sterile and fertile buds, indicating that these are not closely related to Chinese cabbage GMS. Others (*BrEST10704, BrATA7*, and *BrbHLH*) were specifically expressed in fertile buds or up-regulated after F2 buds, implying possible involvement in pollen fertility (Figure 3A). *BrAG* (*Agamous*) determining organ identity was expressed in all seven floral buds, suggesting that it might not be critical in our GMS (Figure 3B). Except for *BrMYB33, BrNAC25*, and *BrASK2*, most genes associated with pollen development in *Arabidopsis* might not be associated with Chinese cabbage GMS determination (Figure 3B). On the other hand, most genes which are related to tapetum specific, pollen coat, pollen wall, kinases, transport, and so on, were specifically expressed in fertile buds (Figure 3C, 3D), implying that they are directly or indirectly the cause and effect on male fertility.

Counterparts of *Arabidopsis* *CYP98A8*, which was highly expressed in the tapetum and developing pollen, and *SHT*, which was coexpressed with *CYP98A8* [55] in Chinese cabbage in a similar fashion to in *Arabidopsis* , indicated that they are involved in male fertility as well.

In conclusion, most important genes essential for the early stage of microsporogenesis in *Arabidopsis* , including *EXS/EMS1, NZZ/SPL, MS5, MS1, MS2, AMS, bHLH89, MYB103/80 MYB35*, and *MYB65*, were highly expressed at least in S1 and S2 buds, meaning that these are not GMS genes in Chinese cabbage. Instead, a signaling factor(s) or another transcription factor(s) that controls the expression of all these genes would be a better candidate for the GMS gene(s) even though we did not identity it in this study. However, *BrMYB99*, which was specifically expressed in F1 and F2 buds (Figure 3C) could be a putative GMS gene, even though the GMS phenotype was different from that of the *Arabidopsis* mutant [13].

Since pollen development is a complex process regulated by the expression of sense- and antisense transcripts as well as small RNAs [89], more comprehensive molecular and genetic study will be required for elucidating GMS mechanism in Chinese cabbage. In addition, 17 *B* *. rapa* -specific genes had no *Arabidopsis* counterpart genes (Table S5). These included Brapa_ESTC000535, Brapa_ESTC003496, Brapa_ESTC003505, Brapa_ESTC003512, Brapa_ESTC003536, Brapa_ESTC003543, Brapa_ESTC003680, Brapa_ESTC003709, Brapa_ESTC003712, Brapa_ESTC003735, Brapa_ESTC005300, Brapa_ESTC030672, Brapa_ESTC042977, Brapa_ESTC048170, Brapa_ESTC049217, and Brapa_ESTC050778. These genes that were highly and specifically expressed in fertile buds will be important genes to investigate in terms of function.

In conclusion, we identified many genes that are differentially expressed between fertile and sterile buds of Chinese cabbage. Most genes are already known in other male sterile plants, but some are newly identified in Chinese cabbage including 17 novel genes. Expression of core transcription factors involved in pollen development were quite similar to *Arabiodopsis* with exception. Numerous genes controlling pollen wall and pollen coat formation were greatly down-regulated in sterile buds, possibly indirect effect of GMS gene defect. All data suggest that Chinese cabbage GMS might be controlled by genes acting in post-meiotic tapetal development.

## Supporting Information

Figure S1 **Genetic model of the genic multiple-allele inherited male sterile line in Chinese cabbage.** Male sterility could be controlled by three different genes at one locus. *Ms* ^*f*^ *, Ms*, and *ms* represent dominant restorer, dominant sterile, and recessive fertile genes, respectively. Correlation of dominance and recessiveness among these genes is *Ms* ^*f*^>*Ms*>*ms*. Dotted boxes indicate plants used in this study.
(DOC)

Figure S2 **The position of probes for each gene in the Br300K Microarray GeneChip.** One hundred and fifty base pairs, occupied by 7 × 60 bp probes with 15 bp overlap, including 60 bp coding sequences and 90 bp 3'-UTR. Otherwise, the 3' 150 bp of non-3' UTR-containing genes were used.
(DOCX)

Figure S3 **Flower structure of fertile and sterile Chinese cabbage used in this study.**
(DOCX)

Figure S4 **Floral buds from fertile and sterile (GMS) Chinese cabbage plants and sample collection.**
(DOCX)

Figure S5 **Analysis of *B* *. rapa* genes used in the Br300K microarray.** A, Comparison of amino acid sequences of *B* *. rapa* to those of other plants. B, Comparison of nucleotide sequences of *B* *. rapa* to those of *Arabidopsis* .
(DOCX)

Figure S6 **Semi-quantitative RT-PCR results from genes showing the highest PI value in each floral bud.** S1-S3 and F1-F4 on the left of each panel expressed floral buds.
(DOC)

Figure S7 **Hierarchical cluster display of the *POD*, *PAP*, and *MATE efflux* genes in Chinese cabbage.** The color scale bar shown above the cluster indicates the maximum and minimum brightness values that represent the PI value.
(DOCX)

Figure S8 **Hierarchical cluster display of *CYP* genes in Chinese cabbage.** The color scale bar shown above the cluster indicates the maximum and minimum brightness values that represent the PI value.
(DOC)

Figure S9 **Hierarchical cluster display of the *LTP family*, *Cys-proteinase*, and carbon supply-related genes in Chinese cabbage.** The color scale bar shown above the cluster indicates the maximum and minimum brightness values that represent the PI value.
(DOC)

Table S1 **Primer sequences used in semi-qRT-PCR.**
(DOCX)

Table S2 **Comparison between fertile and sterile flowers of Chinese cabbage used in this study (unit: mm).** The values are expressed as mean and standard deviation of 10 randomly selected flowers.
(DOC)

Table S3 **Microarray data expressed as PI values.** S1-3 and F1-4 indicate sterile buds 1–3 and fertile buds 1–4, respectively. PI values are expressed as the mean of two independent experiments.
(XLSX)

Table S4 **Number of genes expressed over 2-fold in either sterile or fertile buds.**
(DOCX)

Table S5 **List of specifically expressed genes in fertile buds that were initially classified as no hit found (NHF).** All sequences were subjected to a repeated BLASTn search in NCBI.
(XLSX)

Table S6 **List of specifically expressed genes in sterile buds that were initially classified as no hit found (NHF).** All sequences were subjected to a repeated BLASTn search in NCBI.
(XLSX)

Table S7 **List of genes showing the highest PI values in each floral bud and the primer sequence used in semi-qRT-PCR.**
(DOCX)

Table S8 **Genes specifically expressed in fertile buds.**
(XLSX)

Table S9 **Genes specifically expressed in sterile buds.**
(XLSX)

Table S10 **Change in expression levels of protein kinase genes.** All values are expressed in terms of the ratio of wild type to mutant, so that positive values indicate depression of gene expression in mutants. Dots represent either no difference or no expression. Data for Chinese cabbage were obtained by recalculation, i.e., mean values are used if there are multiple genes.
(DOC)

Table S11 **Change in expression of transporter genes.** All values are expressed in terms of the ratio of wild type to mutant, so that positive values indicate depression of gene expression in mutants. Dots represent either no difference or no expression. Data for Chinese cabbage were obtained by recalculation, i.e., mean values are used if there are multiple genes.
(DOCX)
