# Supplementary figures and images for: Comprehensive Analysis of Genic Male Sterility-Related Genes in Brassica rapa Using a Newly Developed Br300K Oligomeric Chip
Source: PLoS One. 2013 Sep 11;8(9):e72178. doi: 10.1371/journal.pone.0072178 (PMC3770635; doi:10.1371/journal.pone.0072178)

**Figure S1**


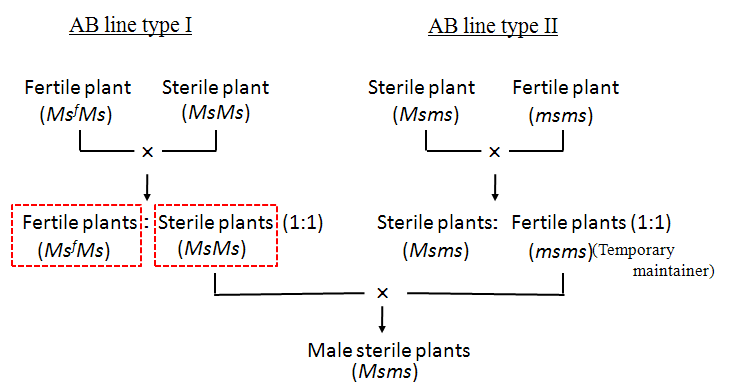

Supplement: Figure S1 — Genetic model of the genic multiple-allele inherited male sterile line in Chinese cabbage. Male sterility could be controlled by three different genes at one locus. Ms f , Ms, and ms represent dominant restorer, dominant sterile, and recessive fertile genes, respectively. Correlation of dominance and recessiveness among these genes is Ms f>Ms>ms. Dotted boxes indicate plants used in this study. (DOC) [file pone.0072178.s001.doc]

**Figure S2**


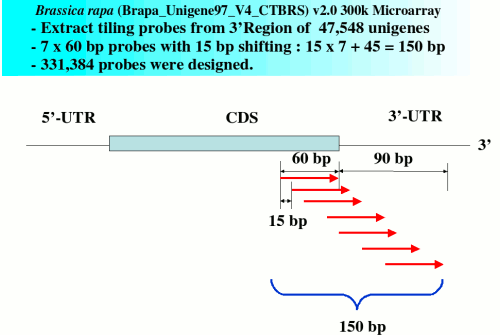

Supplement: Figure S2 — The position of probes for each gene in the Br300K Microarray GeneChip. One hundred and fifty base pairs, occupied by 7 × 60 bp probes with 15 bp overlap, including 60 bp coding sequences and 90 bp 3'-UTR. Otherwise, the 3' 150 bp of non-3' UTR-containing genes were used. (DOCX) [file pone.0072178.s002.docx]

**Figure S3**


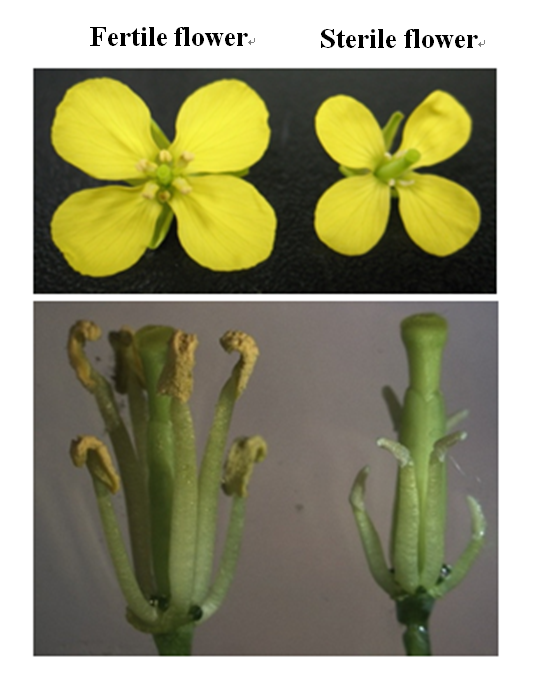

Supplement: Figure S3 — Flower structure of fertile and sterile Chinese cabbage used in this study. (DOCX) [file pone.0072178.s003.docx]

**Figure S4**


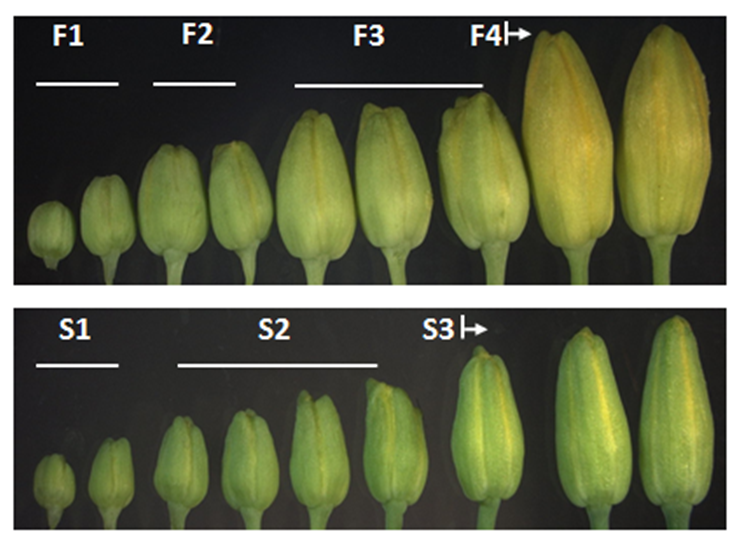

Supplement: Figure S4 — Floral buds from fertile and sterile (GMS) Chinese cabbage plants and sample collection. (DOCX) [file pone.0072178.s004.docx]

**Figure S5**

**A B**


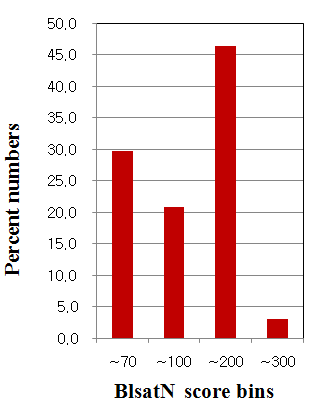

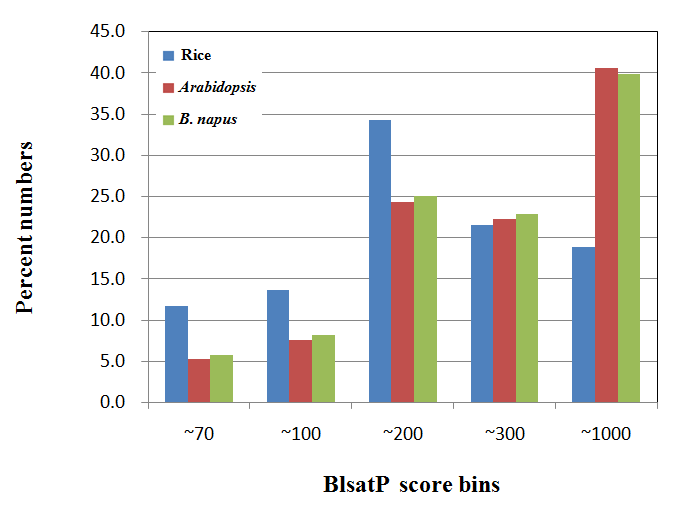

Supplement: Figure S5 — Analysis of B . rapa genes used in the Br300K microarray. A, Comparison of amino acid sequences of B . rapa to those of other plants. B, Comparison of nucleotide sequences of B . rapa to those of Arabidopsis . (DOCX) [file pone.0072178.s005.docx]

**Figure S6**


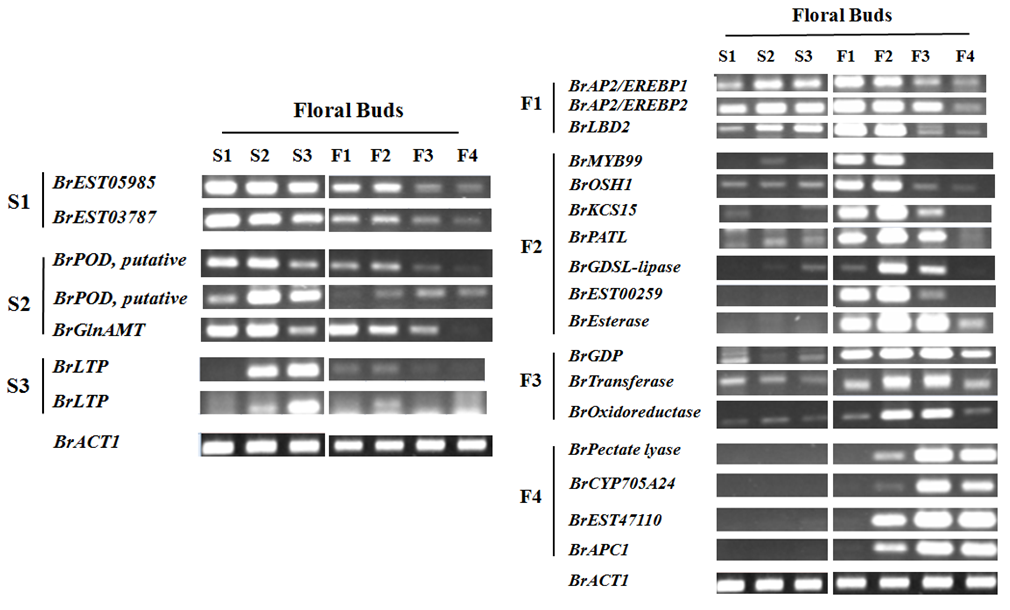

Supplement: Figure S6 — Semi-quantitative RT-PCR results from genes showing the highest PI value in each floral bud. S1-S3 and F1-F4 on the left of each panel expressed floral buds. (DOC) [file pone.0072178.s006.doc]
