# Supplementary material for: Comprehensive Analysis of Genic Male Sterility-Related Genes in Brassica rapa Using a Newly Developed Br300K Oligomeric Chip
Source: PLoS One. 2013 Sep 11;8(9):e72178. doi: 10.1371/journal.pone.0072178 (PMC3770635; doi:10.1371/journal.pone.0072178)
Supplement: Figure S7 — Hierarchical cluster display of the POD, PAP, and MATE efflux genes in Chinese cabbage. The color scale bar shown above the cluster indicates the maximum and minimum brightness values that represent the PI value. (DOCX) [file pone.0072178.s007.docx]

**Figure S7**


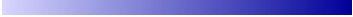


***Arabidopsis***

***B. rapa* sequence Id.**

**PM value**

**F1**

**18,564**

**0**

**S3**

**F4**

**F3**

**F2**

**S2**

**Gene name**

**Gene locus**

**S1**

| **At5g19880** | **Peroxidase, putative** | **Brapa_ESTC029686** |
| --- | --- | --- |
| **At5g64120** | **Peroxidase, putative** | **Brapa_ESTC013772** |
| **At5g64120** | **Peroxidase, putative** | **Brapa_ESTC046528** |
| **At5g06730** | **Peroxidase, putative** | **Brapa_ESTC000533** |
| **At2g41480** | **Peroxidase** | **Brapa_ESTC025135** |
| **At2g41480** | **Peroxidase** | **Brapa_ESTC037341** |
| **At1g68850** | **Peroxidase, putative** | **Brapa_ESTC031639** |
| **At4g17690** | **Peroxidase, putative** | **Brapa_ESTC038768** |
| **At4g17690** | **Peroxidase, putative** | **Brapa_ESTC026013** |
| **At4g17690** | **Peroxidase, putative** | **Brapa_ESTC038050** |
| **At5g47000** | **Peroxidase, putative** | **Brapa_ESTC026030** |
| **At5g47000** | **Peroxidase, putative** | **Brapa_ESTC010986** |
| **At5g47000** | **Peroxidase, putative** | **Brapa_ESTC009283** |
| **At5g47000** | **Peroxidase, putative** | **Brapa_ESTC046132** |
| **At5g47000** | **Peroxidase, putative** | **Brapa_ESTC008076** |
| **At2g01890** | **PAP8** | **Brapa_ESTC030295** |
| **At2g01880** | **ATPAP7/PAP7** | **Brapa_ESTC030294** |
| **At1g14700** | **ATPAP3/PAP3** | **Brapa_ESTC000751** |
| **At1g52940** | **ATPAP5/PAP5** | **Brapa_ESTC020319** |
| **At1g52940** | **ATPAP5/PAP5** | **Brapa_ESTC008653** |
| **At1g56360** | **ATPAP6/PAP6** | **Brapa_ESTC001744** |
| **At2g18130** | **ATPAP11/PAP11** | **Brapa_ESTC041017** |
| **At2g04050** | **MATE efflux family protein** | **Brapa_ESTC023478** |
| **At5g52050** | **MATE efflux protein-related** | **Brapa_ESTC004912** |
| **At4g00350** | **MATE efflux family protein** | **Brapa_ESTC032533** |
| **At4g21900** | **MATE efflux family protein** | **Brapa_ESTC044314** |
| **At4g21900** | **MATE efflux family protein** | **Brapa_ESTC009338** |
| **At3g26590** | **MATE efflux family protein** | **Brapa_ESTC036259** |


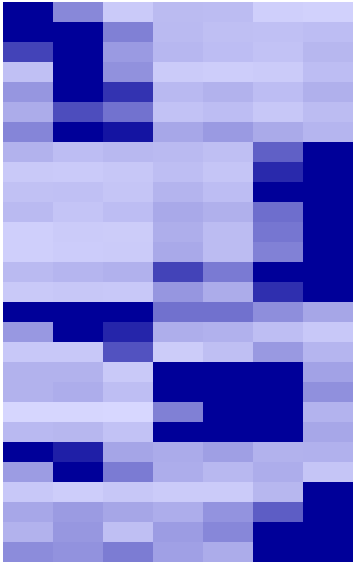


**PAP**

**MATE**

**efflux**

**POD**
