# Supplementary material for: Comprehensive Analysis of Genic Male Sterility-Related Genes in Brassica rapa Using a Newly Developed Br300K Oligomeric Chip
Source: PLoS One. 2013 Sep 11;8(9):e72178. doi: 10.1371/journal.pone.0072178 (PMC3770635; doi:10.1371/journal.pone.0072178)
Supplement: Figure S8 — Hierarchical cluster display of CYP genes in Chinese cabbage. The color scale bar shown above the cluster indicates the maximum and minimum brightness values that represent the PI value. (DOC) [file pone.0072178.s008.doc]

**Figure S8**


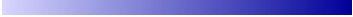


**F1**

**F2**

**F3**

**F4**

**S2**

**S3**

**0**

**19,562**

**PM value**

***Arabidopsis***

***B. rapa* sequence Id.**

**Gene locus**

**Gene name**

**S1**


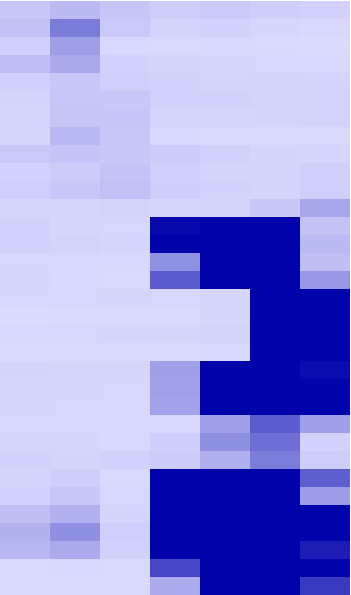


| **At3g26270** | **CYP71B25** | **Brapa_ESTC027924** |
| --- | --- | --- |
| **At3g14620** | **CYP72A8** | **Brapa_ESTC023430** |
| **At3g30290** | **CYP702A8** | **Brapa_ESTC029769** |
| **At2g45570** | **CYP76C2** | **Brapa_ESTC034794** |
| **At3g56630** | **CYP94D2** | **Brapa_ESTC034764** |
| **At5g67310** | **CYP81G1** | **Brapa_ESTC021540** |
| **At1g13080** | **CYP71B2** | **Brapa_ESTC015899** |
| **At5g58860** | **CYP86A1** | **Brapa_ESTC025300** |
| **At2g34500** | **CYP710A1** | **Brapa_ESTC026146** |
| **At5g36220** | **CYP91A1, CYP81D1** | **Brapa_ESTC006219** |
| **At5g36220** | **CYP91A1, CYP81D1** | **Brapa_ESTC023113** |
| **At1g13080** | **CYP71B2** | **Brapa_ESTC039531** |
| **At1g13140** | **CYP86C3** | **Brapa_ESTC015676** |
| **At1g13140** | **CYP86C3** | **Brapa_ESTC018300** |
| **At1g13150** | **CYP86C4** | **Brapa_ESTC017588** |
| **At1g13150** | **CYP86C4** | **Brapa_ESTC039536** |
| **At1g28430** | **CYP705A24** | **Brapa_ESTC007958** |
| **At1g28430** | **CYP705A24** | **Brapa_ESTC049232** |
| **At1g28430** | **CYP705A24** | **Brapa_ESTC007898** |
| **At1g28430** | **CYP705A24** | **Brapa_ESTC049231** |
| **At3g26125** | **CYP86C2** | **Brapa_ESTC025853** |
| **At3g26125** | **CYP86C2** | **Brapa_ESTC017240** |
| **At3g26125** | **CYP86C2** | **Brapa_ESTC016934** |
| **At5g38450** | **CYP735A1** | **Brapa_ESTC027079** |
| **At5g45340** | **CYP707A3** | **Brapa_ESTC003302** |
| **At5g45340** | **CYP707A3** | **Brapa_ESTC006382** |
| **At1g74540** | **CYP98A8** | **Brapa_ESTC007929** |
| **At1g74540** | **CYP98A8** | **Brapa_ESTC017210** |
| **At1g74540** | **CYP98A8** | **Brapa_ESTC038004** |
| **At1g74540** | **CYP98A8** | **Brapa_ESTC028799** |
| **At1g74540** | **CYP98A8** | **Brapa_ESTC010555** |
| **At2g19070** | **Transferase family protein** | **Brapa_ESTC015756** |
| **At2g19070** | **Transferase family protein** | **Brapa_ESTC008208** |
