# Supplementary material for: Comprehensive Analysis of Genic Male Sterility-Related Genes in Brassica rapa Using a Newly Developed Br300K Oligomeric Chip
Source: PLoS One. 2013 Sep 11;8(9):e72178. doi: 10.1371/journal.pone.0072178 (PMC3770635; doi:10.1371/journal.pone.0072178)
Supplement: Figure S9 — Hierarchical cluster display of the LTP family, Cys-proteinase, and carbon supply-related genes in Chinese cabbage. The color scale bar shown above the cluster indicates the maximum and minimum brightness values that represent the PI value. (DOC) [file pone.0072178.s009.doc]

**Figure S9**


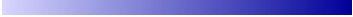


**F1**

**F2**

**F3**

**F4**

**S2**

**S3**

**0**

**19,883**

**PM value**

***Arabidopsis***

***B. rapa* sequence Id.**

**Gene locus**

**Gene name**

**S1**


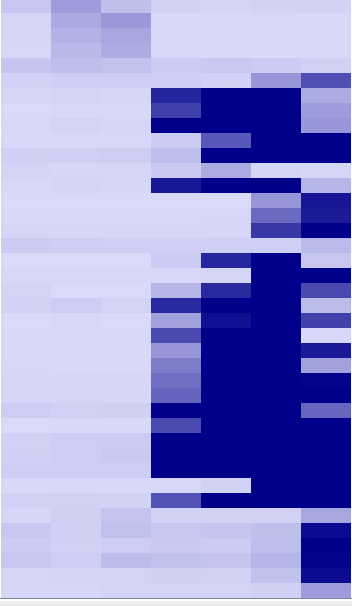


| **At3g22620** | **LTP family protein** | **Brapa_ESTC013008** |
| --- | --- | --- |
| **At2g18370** | **LTP family protein** | **Brapa_ESTC005777** |
| **At2g18370** | **LTP family protein** | **Brapa_ESTC041001** |
| **At2g18370** | **LTP family protein** | **Brapa_ESTC000007** |
| **At4g00165** | **LTP family protein** | **Brapa_ESTC007074** |
| **At1g18280** | **LTP family protein** | **Brapa_ESTC038265** |
| **At3g51590** | **LTP12 protein** | **Brapa_ESTC049901** |
| **At3g51590** | **LTP12 protein** | **Brapa_ESTC001664** |
| **At3g51590** | **LTP12 protein** | **Brapa_ESTC000864** |
| **At4g08670** | **LTP family protein** | **Brapa_ESTC004354** |
| **At4g08670** | **LTP family protein** | **Brapa_ESTC026462** |
| **At4g14815** | **LTP family protein** | **Brapa_ESTC034888** |
| **At4g14815** | **LTP family protein** | **Brapa_ESTC004330** |
| **EU082008** | **LTP-like protein (MF15bh)** | **Brapa_ESTC028019** |
| **EU082018** | **LTP-like protein (MF15yde)** | **Brapa_ESTC049769** |
| **EU082023** | **LTP-like protein (MF15) gene** | **Brapa_ESTC028634** |
| **At4g12545** | **LTP family protein** | **Brapa_ESTC043367** |
| **X60318** | **Phospholipid transfer proteins** | **Brapa_ESTC003598** |
| **At1g66850** | **LTP family protein** | **Brapa_ESTC038086** |
| **AB010433** | **Lipid transfer protein** | **Brapa_ESTC003809** |
| **EU118787** | **E2B-like protein** | **Brapa_ESTC000259** |
| **At1g75930** | **EXL6 protein** | **Brapa_ESTC003525** |
| **At1g20132** | **Hydrolase,** | **Brapa_ESTC047743** |
| **At1g06260** | **Cysteine proteinase, putative** | **Brapa_ESTC027975** |
| **At1g06260** | **Cysteine proteinase, putative** | **Brapa_ESTC010620** |
| **At1g06260** | **Cysteine proteinase, putative** | **Brapa_ESTC018323** |
| **At1g06260** | **Cysteine proteinase, putative** | **Brapa_ESTC010932** |
| **At1g06260** | **Cysteine proteinase, putative** | **Brapa_ESTC038116** |
| **At2g31980** | **Cysteine proteinase inhibitor-related** | **Brapa_ESTC000515** |
| **At2g31980** | **Cysteine proteinase inhibitor-related** | **Brapa_ESTC017953** |
| **At2g31980** | **Cysteine proteinase inhibitor-related** | **Brapa_ESTC027150** |
| **At2g31980** | **Cysteine proteinase inhibitor-related** | **Brapa_ESTC013587** |
| **At2g31980** | **Cysteine proteinase inhibitor-related** | **Brapa_ESTC007955** |
| **At2g31980** | **Cysteine proteinase inhibitor-related** | **Brapa_ESTC000762** |
| **At4g36880** | **Cysteine proteinase, putative** | **Brapa_ESTC027007** |
| **At3g52600** | **CWINV2 protein** | **Brapa_ESTC034099** |
| **At3g52600** | **CWINV2 protein** | **Brapa_ESTC009236** |
| **At3g52600** | **CWINV2 protein** | **Brapa_ESTC027284** |
| **At3g52600** | **CWINV2 protein** | **Brapa_ESTC009304** |
| **At3g52600** | **CWINV2 protein** | **Brapa_ESTC005384** |

***LTP***

***Cys proteinase***

**Carbon supply**
