# Supplementary material for: Comprehensive Analysis of Genic Male Sterility-Related Genes in Brassica rapa Using a Newly Developed Br300K Oligomeric Chip
Source: PLoS One. 2013 Sep 11;8(9):e72178. doi: 10.1371/journal.pone.0072178 (PMC3770635; doi:10.1371/journal.pone.0072178)
Supplement: Table S1 — Primer sequences used in semi-qRT-PCR. (DOCX) [file pone.0072178.s010.docx]

| ***B. rapa* SEQ_ID** | ***At* Locus** | **Description** | **Primer sequences** | |
| --- | --- | --- | --- | --- |
|  |  |  | **Forward** | **Reverse** |
| Brapa_ESTC020996 | At4g27330 | SPOROCYTELESS/NOZZLE (SPL) | GCTGCAAGGCTTTCCAAGCTAC | TATTTCGAAAACCCACCACCGT |
| Brapa_ESTC043424 | At4g20900 | Male sterility MS5; pollenless3 | GGAGAGCTTTGTGTTTGGAGCC | GAAGGAGGAACATGCGAGTGGT |
| Brapa_ESTC010704 | At1g61110 |  | CTAAGCCACCCGACTCAACCAC | AAACGGCCATACCATCGAAAAA |
| Brapa_ESTC029822 | At5g07280 | EXCESS MICROSPOROCYTES1/EXTRASPOROGENOUS CELLS (EMS1) | CATCCACAGAGACATCAAGGCG | ACACGTAGCATGGCCTGTTTGA |
| Brapa_ESTC015748 | At3g42960 | Alcohol dehydrogenase (ATA1) | GTAAACAAACTTGTTTCGGTCAATGTG | CGGTCAGTAAACTTCCTTTCTCAGC |
| Brapa_ESTC011088 | At4g28395 | *Arabidopsis thaliana* anther 7 (ATA7) | GAAGAATGAGCAGGGGACTGGA | CGTAGCCCGGGCAAAGTATCTA |
| Brapa_ESTC001042 | At3g11980 | Male sterility protein 2 (MS2) | ACCAAAGCAATGGGGAAATTCA | ATCTGCCTTAGCCATTCCATGC |
| Brapa_ESTC001024 | At4g14080 | Glycosyl hydrolase family 17 protein / anther-specific protein (A6) | ATTATGGAATCCTGCATCCCGA | TTTTCGGTACGAAACTCAGCCC |
| Brapa_ESTC003402 | At3g23150 | ETR2 | TCACTGCCATTGCACCTAGCTC | CCGGCTTTCTCACAACTCCATT |
| Brapa_ESTC006268 | At4g31500 | CYP83B1 | CGAGGGTCAGGCAGAAGAATGT | CCTGATCAAATGTGCGTCCTTG |
| Brapa_ESTC007874 | At1g71770 | Polyadenylate-binding protein 5 (PAB5) | ACAGGATGAACGGAGTGGAAGC | TTGAGGAGGGAGCAAACTGGTC |
| Brapa_ESTC000961 | At1g69500 | Cytochrome P450 | AGGCCGAAGAGACAAACACACC | ACTTGAATGGGGAAGCGTTCTG |
| Brapa_ESTC015754  Brapa_ESTC044174  Brapa_ESTC010964  Brapa_ESTC036883  Brapa_ESTC037115  -  Brapa_ESTC046330  Brapa_ESTC016224  Brapa_ESTC023372  Brapa_ESTC010368  Brapa_ESTC010704  Brapa_ESTC031607 | At1g06170  At4g18960  At2g16910  At3g11440  At3g28470  At5g06100  At5g56110  At3g09090  At5g13390  At5g57800  At1g61110  At3g61160 | Basic helix-loop-helix (bHLH) family protein  AGAMOUS (AG)  ABORTED MICROSPORES (AMS)  Myb domain protein 65 (MYB65)  Myb domain protein 35 (MYB35)  Myb domain protein 33 (MYB33)  Myb domain protein 103/80 (MYB103/80)  DEFECTIVE IN EXINE FORMATION 1 (DEX1)  NO EXINE FORMATION (NEF1)  FACELESS POLLEN-1 (FLP1)  ANAC025 (NAC25)  ASK-beta (ASK2) | CTCCAAAGTGATTGATCAGCTTCAG  GGGTCAATGTCTCCCAAAGAGCT  GGAGCTTCAAGACGAGCTTGAAGA  GAGCTCCCTTCATTCCAATATTCAGA  ACATTAGCGGCCATGGAAACTC  GAYAAYGAGATAAAGAAYTATTGGAACAC  CTGTCCTTGAAGGAATGTTCTCTCAA  AGGAGGATAAAGCAGAGCCAGATCTAT  CTCTAATCCCAATCAAATACGTGGTAGAA  CTTGGATCGTTGGGAAATGGTT  TTTTTCGATGGTATGGCCGTTT  CTGGAGAGACTAGTGTTGATCAATTGGT | ACTAGCTTCAAGCCCACATAGATAAAAC  GTGGCATGATCTGCTCGTAGTTAG  CATTTGTAACCTCCAGCCCCAA  ACCCGCACTAAGTTGCGTGG  CTGCGCAAAAGTGAGGTTGCTA  CAGAAGCTCCAGGTAACAAGCTGG  CATCTTGAACACTGTTGTCAAGTGGA  CGAGAAGGATGGTAAGGGAACG  CCACGCAACATTATCACTGAGAGTT  GCAGGTTCCTAGTCCTTGAACATCTTT  TAGGGAGCTGAAACGCCTGTCT  CTTCCATTAGGCAAGGATGCTCTC |
| Brapa_ESTC008149 | At1g75910 | Extracellular lipase;anther-specific proline-rich-like protein (APG-like) | CGAGTCCTTGCATAACCGTGTG | TCCGGACATGTTCATGCATTCT |
| Brapa_ESTC011474 | At5g07550 | Glycine-rich protein GRP 19 (PUTG-1) | TCCGCCGGTTATCAAAAGATGT | CCCCCTTCCGTAGTTGGATTTT |
| Brapa_ESTC028646 | At5g07560 | Oleosin-like protein GRP 20 (ATGRP4) | CCTCTTGGTTCTTGCCGGTTTA | AGACCTGATGTTTGGGCAGCTC |
| Brapa_ESTC003556 | At1g20120 | Anther-specific proline-rich protein APG precursor | GGACAGGGATGGTCGAATTAGG | CTCAACTAATGTTCCAAAAAATACATTAAACC |
| Brapa_ESTC007739 | At1g75940 | Betaglucosidase | ACCTTCAGTCGTGCCAACTTCC | CGTGGTAAAACTGCACACCTGC |
| Brapa_ESTC027006 | At2g25890 | Glycine-rich protein/oleosin | TTGTGCCTGCGGTGATAACAAT | AGGAGCTGTTGTGGTTTGGTCC |
| Brapa_ESTC020348 | At1g61110 | NAM prtoein | ACTACCGGTCTCAATCATCGCC | AACACAAGCGCTTTCTTGACCC |
| Brapa_ESTC028843 | At5g62320 | MYB DOMAIN PROTEIN 99 (MYB99), | AGGATGTTCTTCGGTGGGAGAG | GTACAAAGCCTTAATTCGTTCAGTGCT |
| Brapa_ESTC010620 | At1g06260 | Cysteine proteinase | AGCCTAGAAGTCGCTGCTGCTC | TAGCCTTCTTCACCCCAACCAG |
| Brapa_ESTC010469 | At1g68875 | Expressed protein | CACAGAAAGCCAAAATGGCAGA | TCAGCGGTTTGCAGTACATGGT |
| Brapa_ESTC000987 | At4g37900 | Putative protein | TAGAGCCGTGGGGTTGCTAGAC | GCTCCTTGGACAAGCACCTAGC |
| Brapa_ESTC030090 | At1g23570 | Hypothetical prtein | AAACGGTTGTGCAAAAAGAGGC | ATCAAAGCCATTTCCTGAAGCG |
| Brapa_ESTC019311 | At2g31500 | Putative calcium-dependent protein kinase | GTGTTCCTCCTTTTTGGGCAGA | CCGTTCTTGTCAGTGTCCATCG |
| Brapa_ESTC013633 | At3g20530 | Protein kinase family protein | GTGCGGAACAAGAAGAAACCGT | CGACACATGAGTCTCTCCACCG |
| Brapa_ESTC009360 | At3gg01085 | Protein kinase family protein | TCCTGAGAAACGAGGAACTGCC | AAACCAGTGTACGGGAACTCGC |
| Brapa_ESTC009271 | At5g15110 | Polysaccharide lyase family 1 (pectate lyase) | CGATTGATAAATGCTGGCGTTG | CGATATGGACATCAGCTCCACG |
| Brapa_ESTC009278 | At3g42640 | Plasma membrane H+-ATPase-like protein | AATGGTATCGGAGCAGCAAAGG | CTACGCCCGGTCTCTATTCCAA |
| Brapa_ESTC007963 | At1g80660 | Plasma membrane ATPase, putative | CTGAACGTCCTGGCTTCTGGTT | CGTGAAAGCGGTCTTGTTCTCA |
| Brapa_ESTC027348 | At2g13680 | Callose synthase 5 (CALS5) | AGAAGTTTAGCGCGGATTTCCA | TCTGAAATTCGGAGACGAAGGG |
| Brapa_ESTC027135 | At5g22260 | MALE STERILITY 1 (MS1) | ATATTCTGCACAATCGCGTGGA | TCTCCATCATCCTCCTTCGCTC |
| Brapa_ESTC000842 | At1g20130 | Family II extracellular lipase, putative (APG) | AGTGATCGGAACACCACCGATT | CTCTGACTGGGATGGAGACCGT |
| Brapa_ESTC018054 | At5g61610 | Glycine-rich protein / oleosin | AGCTTCTGGTAGGCTTGGAGCA | CGCTTGGAGCCAATTGAATTTT |
| Brapa_ESTC001657 | At5g07600 | Glycine-rich protein / oleosin (Oleosin) | TTGCCTCCGTGGTCTTTTTAGG | TCCTTCCTTGCCAGGATGCTTA |
| Brapa_ESTC008102 | At1g23240 | Caleosin-related family protein | CAAGAAGCATGCTCGCTCACAT | TGGATGAATGGGCTTTGGAAAT |
| Brapa_ESTC010555 | At1g74540 | CYP98A8 | GGCAGAGATGATCAGATGCCCT | TCCAAACGGAAGAACCCTGAAG |
| Brapa_ESTC015756 | At2g19070 | SPERMIDINE HYDROXYCINNAMOYL TRANSFERASE (SHT) | TCTGGACTGGTAAACCGCTTCC | GGCTGCACCCGACTACGAGTAT |
| Bra037560* | AT1G49240 | Member of a subclass of actins composed of ACT2 | GAACCGGGTGCTCCTCAGGA | ATGGTACCGGAATGGTCAAGGC |
| * Asterisks indicate gene ID in BGI database (http://brassicadb.org) | | | | |

**Table S1**. Primer sequences used in semi-qRT-PCR.
