# Supplementary material for: Comprehensive Analysis of Genic Male Sterility-Related Genes in Brassica rapa Using a Newly Developed Br300K Oligomeric Chip
Source: PLoS One. 2013 Sep 11;8(9):e72178. doi: 10.1371/journal.pone.0072178 (PMC3770635; doi:10.1371/journal.pone.0072178)
Supplement: Table S2 — Comparison between fertile and sterile flowers of Chinese cabbage used in this study (unit: mm). The values are expressed as mean and standard deviation of 10 randomly selected flowers. (DOC) [file pone.0072178.s011.doc]

**Table S2**. Comparison between fertile and sterile flowers of Chinese cabbage used in this study (unit: mm). The values are expressed as mean and standard deviation of 10 randomly selected flowers.

|  | Sepal length | Petal length | Petal  width | Long stamen  length | Short stamen length | Pistil length | Pistil diameter |
| --- | --- | --- | --- | --- | --- | --- | --- |
| Fertile flower | 5.88±0.23^**^ | 10.79±0.76^**^ | 6.61±0.42^**^ | 7.04±0.30^**^ | 4.91±0.46^**^ | 6.37±0.21^**^ | 1.11±0.05 |
| Sterile flower | 5.23±0.52 | 8.91±0.38 | 5.42±0.26 | 3.95±0.40 | 2.17±0.51 | 5.93±0.34 | 1.08±0.07 |

^**^ show significance level at 1% of T test
