# Supplementary material for: Comprehensive Analysis of Genic Male Sterility-Related Genes in Brassica rapa Using a Newly Developed Br300K Oligomeric Chip
Source: PLoS One. 2013 Sep 11;8(9):e72178. doi: 10.1371/journal.pone.0072178 (PMC3770635; doi:10.1371/journal.pone.0072178)
Supplement: Table S4 — Number of genes expressed over 2-fold in either sterile or fertile buds. (DOCX) [file pone.0072178.s013.docx]

**Table S4.**  Number of genes expressed over 2-fold in either sterile or fertile buds..

| Over 2-fold up-regulated genes in sterile buds  [5,848] ^a^ | | | | Over 2-fold up-regulated genes in fertile buds  [4,774] ^b^ | | | |
| --- | --- | --- | --- | --- | --- | --- | --- |
| S1/F1 | S2/F2 | S3/F3 | S3/F4 | F1/S1 | F2/S2 | F3/S3 | F4/S3 |
| 712  (146) | 2,309  (346) | 2,408  (298) | 2,645  (402) | 1,063  (135) | 1,862  (306) | 2,495  (415) | 2,785  (418) |

( ): no hit (no match to *Arabidopsis* gene)

^a^ Actual number will be 3,451 because 28% of them are overlapped.

^b^ Actual number will be 3,438 because 41% of them are overlapped.
