# Supplementary material for: Comprehensive Analysis of Genic Male Sterility-Related Genes in Brassica rapa Using a Newly Developed Br300K Oligomeric Chip
Source: PLoS One. 2013 Sep 11;8(9):e72178. doi: 10.1371/journal.pone.0072178 (PMC3770635; doi:10.1371/journal.pone.0072178)
Supplement: Table S7 — List of genes showing the highest PI values in each floral bud and the primer sequence used in semi-qRT-PCR. (DOCX) [file pone.0072178.s016.docx]

| ***Br* SEQ_ID** | ***At* Locus** | **Description** | **Primers** | |
| --- | --- | --- | --- | --- |
|  |  |  | **Forward** | **Reverse** |
| Brapa_ESTC005985 | At2g36430 | Unknown | AGTTTCTTTCCGAACTGCGTGG | CTAGGTCGAACGCCACGTCTCT |
| Brapa_ESTC003787 | At2g43720 | Unknown | TTAGCCAATGCGCAGAGACTTG | TTTGACACGAGACTAGGCAGCG |
| Brapa_ESTC013772 | At5g64120 | Peroxidase, putative | ACAATGTCCCCAAAACGGTGAT | TTAATTAACCGCGGAGCAAACC |
| Brapa_ESTC013027 | At5g06720 | Peroxidase, putative | CAAAATTTTCAGCTGTCGGGCT | GTTATCGAACGCGTCAGGTGTG |
| Brapa_ESTC013518 | At1g15040 | Glutamin amidotransferase (AMT) | GAGCTGCCAGGACTCTCTCAGG | TGATCTGATCCACCTCGTCGAA |
| Brapa_ESTC005777 | At2g18370 | Lipid transferase protein (LTP) | ACCTCGTTATTCCGAATCTGCG | AGGGCTTTAGCCAATTCAGGCT |
| Brapa_ESTC041001 | At2g18370 | Lipid transferase protein (LTP) | ACCTCGTTATTCCGAATCTGCG | GACTTGATGCATTGACAAGCCG |
| Brapa_ESTC002858 | At1g12610 | AP2/EREBP1 transcription factor | TTTCAGAGGTATACGGCGGAGG | ATGACATCCGGGTGGTTTGACT |
| Brapa_ESTC005523 | At5g51990 | AP2/EREBP1 transcription factor | GCTTCGAGCTGTCCGAAGAAAC | GCGAAATTAAGACAAGCGGAGC |
| Brapa_ESTC025479 | At1g06280 | LOB domain-containing protein 2 (LBD2) | GTACGTGAAGAGGACCGCCCTA | TGCTTTAAATTTCCCCAGCCAC |
| Brapa_ESTC028843 | At5g62320 | MYB99 | GGATGTTCTTCGGTGGGAGAGC | GCCTTAATTCGTTCAGTGCTTTAATATCT |
| Brapa_ESTC032819 | At5g01580 | Oas high accumulation 1 (OSH1); thiol reductase | AAAACAAGCTCGAACAATGGGC | TGGCAACTGGTTGCTTCCATAA |
| Brapa_ESTC017911 | At3g52160 | 3-Ketoacyl-CoA synthase 15 (KCS15) | CTCCTTCATCCTCTGGTCTTCCTC | GCCATCGCGGAGATTCACTT |
| Brapa_ESTC034136 | At4g37050 | Patatin-like protein (PATL) | CTCTTCTTACCAGGCGGTGAGC | CGAACCATCTGCACAGATCCAG |
| Brapa_ESTC025896 | At2g03980 | GDSL-motif lipase/hydrolase family protein | ATCATTGCAGAGACGGTACCCC | TACACGCCTTTGAGAATGGCAA |
| Brapa_ESTC000259 | no_hits_found | Unknown | GCTTGTGGGCACACTCCCTACT | TACAACAGCGGTTACGGCTTCA |
| Brapa_ESTC018145 | At3g26820 | Esterase/lipase/thioesterase family protein | TGTCCATCAAATCGGTGGAATG | TGTCTCCCATGTTCAGAAGCCA |
| Brapa_ESTC014952 | At5g08030 | Glycerolphosphoryl diester phosphodiesterase (GDP) | TCAGCCATGGTCGTCAAGTGTT | AATCTGCACCTTCCTCAATCGC |
| Brapa_ESTC003644 | At2g25150 | Transferase | TAGCACCTCCTCGACATCCCTC | AAGCGAAACCCCAAAGGAGAGT |
| Brapa_ESTC027921 | At4g10500 | Oxidoreductase, 2OG-Fe(II) oxygenase family | GAACACCGAGAAGGAGCGGTTA | TGGAAACCGTGTAGTGGTGGAA |
| Brapa_ESTC007723 | At1g30350 | Pectate lyase | GCAATGTTGCTCGGACATTCTG | TTTTGCGGCAGGACTTTTATCC |
| Brapa_ESTC007898 | At1g28430 | CYP705A24 | TGGAGGAACATCAAGGTGCTGA | CCTTCTTGAAACTCCCTTGGCA |
| Brapa_ESTC047110 | no_hits_found | Unknown | AAACCAACTTGCAAGCAGACCC | TGTTGGACTGCAAATAAGCATGTG |
| Brapa_ESTC045786 | At5g17480 | Arabidopsis pollen calcium-binding protein (APC1) | ATGGCTGATGCTGAGCACGA | CTAGAAAACTTTGGCAACATCCTTCA |

**Table S7**. List of genes showing the highest PI values in each floral bud and the primer sequence used in semi-qRT-PCR.
