# Supplementary material for: Comprehensive Analysis of Genic Male Sterility-Related Genes in Brassica rapa Using a Newly Developed Br300K Oligomeric Chip
Source: PLoS One. 2013 Sep 11;8(9):e72178. doi: 10.1371/journal.pone.0072178 (PMC3770635; doi:10.1371/journal.pone.0072178)
Supplement: Table S10 — Change in expression levels of protein kinase genes. All values are expressed in terms of the ratio of wild type to mutant, so that positive values indicate depression of gene expression in mutants. Dots represent either no difference or no expression. Data for Chinese cabbage were obtained by recalculation, i.e., mean values are used if there are multiple genes. (DOC) [file pone.0072178.s019.doc]

Table S10. Change in expression levels of protein kinase genes. All values are expressed in terms of the ratio of wild type to mutant, so that positive values indicate depression of gene expression in mutants. Dots represent either no difference or no expression. Data for Chinese cabbage were obtained by recalculation, i.e., mean values are used if there are multiple genes.

| ***At* Locus** | **Description** | **F1/S1** | **F2/S2** | **F3/S3** | **F4/S3** | ***B. rapa* SEQ_ID** |
| --- | --- | --- | --- | --- | --- | --- |
| At4g00970 | Protein kinase family protein | -4.5 | -12.4 | -3.3 | -4.7 | Brapa_ESTC026282 |
| At4g04570 | Protein kinase family protein | -2.0 | -5.1 | -1.6 | -1.8 | Brapa_ESTC004917 |
| At5g60270 | Lectin Protein kinase family protein | -4.2 | -7.2 | -4.6 | -4.4 | Brapa_ESTC034973 |
| At1g14370 | APK2A (Protein kinase 2A) | -2.5 | -4.5 | -2.3 | -2.9 | Brapa_ESTC005206 |
| At1g72540 | Protein kinase, putative | -2.6 | -9.7 | -1.6 | -1.1 | Brapa_ESTC014795 |
| At3g21910 | Receptor-like Protein kinase-related | 60.0 | 110.9 | 217.3 | 44.6 | Brapa_ESTC017477 |
| At1g04700 | Protein kinase family protein | 1.2 | 1.3 | 1.1 | 4.3 | Brapa_ESTC033230 |
| At1g10620 | Protein kinase family protein | -1.2 | -1.4 | 1.5 | 27.6 | Brapa_ESTC035855, 27273 |
| At1g11340 | S-locus lectin Protein kinase family protein | 5.2 | 4.5 | 1.0 | -1.1 | Brapa_ESTC019429 |
| At1g16760 | Protein kinase family protein | -1.0 | -1.7 | 1.9 | 26.7 | Brapa_ESTC017193, 39636 |
| At1g23540 | Protein kinase family protein | -1.2 | 1.2 | 8.6 | 10.5 | Brapa_ESTC030089 |
| At1g23540 | Protein kinase family protein | 1.8 | 1.1 | 4.9 | 47.5 | Brapa_ESTC027361 |
| At1g50990 | Protein kinase-related | 2.9 | 35.0 | 44.6 | 23.3 | Brapa_ESTC040363 |
| At1g61860 | Protein kinase, putative | 2.5 | 1.2 | 3.6 | 41.1 | Brapa_ESTC019382 |
| At1g72460 | Leucine-rich repeat transmembrane Protein kinase, putative | -1.5 | -1.3 | 3.8 | 22.2 | Brapa_ESTC040513 |
| At1g76370 | Protein kinase, putative | -2.0 | -1.2 | 3.4 | 37.7 | Brapa_ESTC019375, 40553 |
| At2g16750 | Protein kinase family protein | 3.0 | 8.9 | 4.6 | 11.0 | Brapa_ESTC037083, 16919 |
| At2g21480 | Protein kinase family protein | 4.4 | 6.8 | 4.6 | 40.8 | Brapa_ESTC050378, 50377 |
| At2g31500 | CPK24 (calcium-dependent Protein kinase 24) | 3.6 | 1.5 | 9.2 | 79.4 | Brapa_ESTC027298, 27341, 41393, 28999, 10825, 19311 |
| At2g38910 | CPK20 (calcium-dependent Protein kinase 20) | -1.2 | -1.1 | 1.4 | 11.8 | Brapa_ESTC042024 |
| At2g41970 | Protein kinase, putative | 13.0 | 15.3 | 2.2 | 23.9 | Brapa_ESTC041966 |
| At2g43230 | Serine/threonine Protein kinase, putative | 1.8 | -1.4 | 5.2 | 29.5 | Brapa_ESTC050347, 27325, 09349 |
| At3g01085 | Protein kinase family protein | 1.8 | 2.0 | 3.9 | 30.8 | Brapa_ESTC026065, 42396, 09360 |
| At3g02810 | Protein kinase family protein | 2.2 | 1.4 | 3.9 | 37.2 | Brapa_ESTC026044, 42485 |
| At3g05140 | Protein kinase family protein | -1.2 | -1.2 | 1.4 | 24.0 | Brapa_ESTC042593 |
| At3g12690 | Protein kinase, putative | -1.3 | -1.1 | 2.4 | 24.3 | Brapa_ESTC042881 |
| At3g13065 | Leucine-rich repeat transmembrane Protein kinase, putative | 1.3 | -1.4 | 6.4 | 43.7 | Brapa_ESTC019302, 49875, 49876, 42834 |
| At3g15890 | Protein kinase family protein | 1.6 | 1.8 | 3.8 | 1.5 | Brapa_ESTC036298 |
| At3g18810 | Protein kinase family protein | 2.9 | 1.6 | 3.0 | 22.8 | Brapa_ESTC034631, 09246, 19269 |
| At3g20190 | Leucine-rich repeat transmembrane Protein kinase, putative | 1.3 | 1.3 | 1.6 | 10.5 | Brapa_ESTC019246 |
| At3g20200 | Protein kinase family protein | 1.1 | 1.2 | 1.5 | 6.3 | Brapa_ESTC029920 |
| At3g20530 | Protein kinase family protein | -1.4 | 1.0 | 2.4 | 19.3 | Brapa_ESTC029039, 32802, 19234, 13633 |
| At3g21910 | Receptor-like Protein kinase-related | 76.5 | 69.1 | 279.6 | 42.7 | Brapa_ESTC008393, 17133, 25860, 08289 |
| At3g21920 | Pollen coat receptor kinase, putative /receptor-like kinase-related | 58.9 | 157.8 | 145.6 | 61.1 | Brapa_ESTC028841 |
| At3g21930 | Receptor-like Protein kinase-related | 56.7 | 77.1 | 53.0 | 14.2 | Brapa_ESTC003608 |
| At3g21990 | Receptor-like Protein kinase-related | 10.6 | 25.4 | 24.3 | 25.1 | Brapa_ESTC028017 |
| At3g22040 | Receptor-like Protein kinase-related | -1.2 | -1.2 | 29.7 | 66.2 | Brapa_ESTC033231, 17378, 33220, 07964 |
| At3g22050 | Receptor-like Protein kinase-related | 2.0 | -1.9 | 106.7 | 315.6 | Brapa_ESTC003650 |
| At3g24720 | Protein kinase family protein | 1.6 | 1.6 | 1.1 | 10.3 | Brapa_ESTC006454 |
| At3g29040 | Receptor-like Protein kinase-related | 6.1 | 5.1 | 40.2 | 52.0 | Brapa_ESTC033778 |
| At3g47570 | Leucine-rich repeat transmembrane Protein kinase, putative | -1.2 | -1.1 | 6.0 | 2.0 | Brapa_ESTC031864 |
| At3g58310 | Receptor-like Protein kinase-related | -1.4 | 1.4 | 11.0 | 22.8 | Brapa_ESTC021028 |
| At3g59830 | Ankyrin Protein kinase, putative | 1.0 | 1.7 | 3.5 | 34.4 | Brapa_ESTC043910, 27327, 31832 |
| At3g61160 | Shaggy-related Protein kinase beta / ASK-beta (ASK2) | 3.0 | 14.2 | 55.4 | 47.1 | Brapa_ESTC005304, 10492 |
| At4g14780 | Protein kinase, putative | 2.5 | 13.1 | 20.7 | 12.3 | Brapa_ESTC007787 |
| At4g31230 | Protein kinase family protein | 1.0 | 1.3 | 1.0 | 6.9 | Brapa_ESTC019206 |
| At4g34440 | Protein kinase family protein | -1.4 | -1.7 | 3.0 | 32.3 | Brapa_ESTC044991 |
| At5g02110 | Cyclin-dependent Protein kinase (CYCD7;1) | 1.6 | 1.0 | 8.0 | 73.4 | Brapa_ESTC029004, 09348 |
| At5g12180 | CPK17 (calcium-dependent Protein kinase 17) | 2.3 | 1.1 | 4.1 | 20.5 | Brapa_ESTC032129 |
| At5g18910 | Protein kinase family protein | 1.3 | 1.0 | 4.0 | 25.5 | Brapa_ESTC029914, 34502 |
| At5g18910 | Protein kinase family protein | 1.0 | 1.2 | 2.5 | 23.2 | Brapa_ESTC019193 |
| At5g19360 | CPK34 (calcium-dependent Protein kinase 34) | 2.0 | 1.7 | 4.8 | 37.5 | Brapa_ESTC027274 |
| At5g20690 | Leucine-rich repeat transmembrane Protein kinase, putative | -1.1 | -1.2 | -1.2 | 5.9 | Brapa_ESTC045826 |
| At5g57670 | ATP binding / Protein kinase/ protein serine/threonine kinase | 1.3 | 1.6 | 2.9 | 7.2 | Brapa_ESTC005857 |
| At3g56600 | Inositol or phosphatidylinositol kinase | 1.3 | 1.4 | 1.8 | 14.0 | Brapa_ESTC034772 |
| At3g56960 | Phosphatidylinositol-4-phosphate 5-kinase family protein | 1.0 | -1.6 | 3.0 | 36.5 | Brapa_ESTC036110, 36112 |
| At2g24370 | Kinase | 1.7 | 1.0 | 5.4 | 68.5 | Brapa_ESTC019292, 27343 |
| At2g41210 | Phosphatidylinositol-4-phosphate 5-kinase family protein | -1.2 | -1.7 | 5.7 | 96.9 | Brapa_ESTC035948 |
| At5g45820 | CIPK20 (CBL-INTERACTING PROTEIN KINASE 20) | 1.0 | 1.0 | 3.3 | 20.0 | Brapa_ESTC020351, 46043 |
| At3g50390 | Transducin family protein / WD-40 repeat family protein | -1.4 | -1.7 | 1.7 | 12.6 | Brapa_ESTC043396, 33046, 17132 |
| At4g39110 | Protein kinase family protein | 2.0 | 2.3 | 2.7 | 10.7 | Brapa_ESTC019312, 45159 |
| At5g57690 | Diacylglycerol kinase | -1.4 | 1.7 | 2.1 | 8.3 | Brapa_ESTC034685 |
| At5g65530 | Protein kinase, putative | 1.0 | 1.0 | -1.1 | 1.5 | Brapa_ESTC021012, 20692, 24065, 01347, 19385 |
