# Supplementary material for: Comprehensive Analysis of Genic Male Sterility-Related Genes in Brassica rapa Using a Newly Developed Br300K Oligomeric Chip
Source: PLoS One. 2013 Sep 11;8(9):e72178. doi: 10.1371/journal.pone.0072178 (PMC3770635; doi:10.1371/journal.pone.0072178)
Supplement: Table S11 — Change in expression of transporter genes. All values are expressed in terms of the ratio of wild type to mutant, so that positive values indicate depression of gene expression in mutants. Dots represent either no difference or no expression. Data for Chinese cabbage were obtained by recalculation, i.e., mean values are used if there are multiple genes. (DOCX) [file pone.0072178.s020.docx]

**Table S11.** Change in expression of transporter genes. All values are expressed in terms of the ratio of wild type to mutant, so that positive values indicate depression of gene expression in mutants. Dots represent either no difference or no expression. Data for Chinese cabbage were obtained by recalculation, i.e., mean values are used if there are multiple genes.

| ***At* Locus** | **Proposed function** | **F1/S1** | **F2/S2** | **F4/S3** | **F3/S3** | ***B. rapa* SEQ_ID** |
| --- | --- | --- | --- | --- | --- | --- |
| At3g30340 | Nodulin MtN21 family protein | 1.0 | -3.0 | -6.5 | -6.5 | Brapa_ESTC038196, 22363 |
| At4g08300 | Nodulin MtN21 family protein | 1.1 | -1.2 | -2.8 | -3.5 | Brapa_ESTC025944, 21426 |
| At5g40240 | Nodulin MtN21 family protein | -1.6 | -1.3 | -1.9 | -1.6 | Brapa_ESTC045916 |
| At3g11900 | ANT1 (AROMATIC AND NEUTRAL TRANSPORTER 1) | -1.2 | -2.6 | 1.1 | -1.8 | Brapa_ESTC021388 |
| At4g18050 | PGP9 (P-GLYCOPROTEIN 9); ATPase | 1.7 | -1.4 | 4.6 | 3.0 | Brapa_ESTC021871, 17135 |
| At1g02520 | PGP11 (P-GLYCOPROTEIN 11); ATPase, | 2.6 | 15.9 | 9.2 | 32.0 | Brapa_ESTC047747 |
| At1g02530 | PGP12 (P-GLYCOPROTEIN 12); ATPase, | 2.7 | 20.0 | 2.7 | 18.4 | Brapa_ESTC027018 |
| At1g10680 | PGP10 (P-GLYCOPROTEIN 10); ATPase, | -1.1 | 4.9 | 2.4 | 5.7 | Brapa_ESTC035840 |
| At2g07560 | AHA6 (ARABIDOPSIS H(+)-ATPase 6) | -1.3 | 2.5 | 181.4 | 112.1 | Brapa_ESTC042291 |
| At3g42640 | AHA8 (ARABIDOPSIS H(+)-ATPase 8) | 2.3 | 2.2 | 26.1 | 5.8 | Brapa_ESTC026026, 09278 |
| At1g80660 | AHA9 (Arabidopsis H(+)-ATPase 9) | 1.6 | -0.6 | 165.9 | 84.5 | Brapa_ESTC040810, 07963, 20242 |
| At3g08560 | VHA-E2 (VACUOLAR H+-ATPase SUBUNIT E ISOFORM 2) | 2.1 | 1.3 | 340.6 | 85.7 | Brapa_ESTC007883 |
| At3g50930 | AAA-type ATPase family protein | 1.9 | -2.6 | 11.5 | 1.2 | Brapa_ESTC017672 |
| At4g28000 | AAA-type ATPase family protein | -2.1 | 3.1 | 21.0 | 3.3 | Brapa_ESTC037275 |
| At5g44790 | RAN1 (RESPONSIVE-TO-ANTAGONIST1); ATPase | 2.1 | 1.4 | 33.1 | 3.0 | Brapa_ESTC019417 |
| At5g46240 | KAT1 (K+ ATPase 1); cyclic nucleotide binding / inward rectifier potassium channel | -2.2 | -2.1 | 1.1 | -2.6 | Brapa_ESTC046021 |
| At4g32510 | Anion exchanger | 1.2 | 4.2 | 40.8 | 23.9 | Brapa_ESTC044815 |
| At1g16380 | ATCHX1 (CATION/H+ EXCHANGER 1); monovalent cation:proton antiporter | -1.2 | -1.7 | 29.8 | 2.3 | Brapa_ESTC035025 |
| At1g79400 | ATCHX2 (CATION/H+ EXCHANGER 2); monovalent cation:proton antiporter | -1.3 | -1.9 | 20.3 | 2.6 | Brapa_ESTC040713 |
| At2g28180 | ATCHX8 (cation/hydrogen exchanger 8); monovalent cation:proton antiporter | -1.2 | -1.4 | 8.2 | 1.1 | Brapa_ESTC030990 |
| At2g30240 | ATCHX13 (cation/H+ exchanger 13); monovalent cation:proton antiporter | -1.3 | -1.3 | 57.6 | 3.1 | Brapa_ESTC041525 |
| At1g06970 | ATCHX14 (cation/hydrogen exchanger 14); monovalent cation:proton antiporter | -1.3 | -1.4 | 11.0 | -1.1 | Brapa_ESTC030584 |
| At3g17630 | ATCHX19 (CATION/H+ EXCHANGER 19); monovalent cation:proton antiporter | -1.3 | 1.2 | 71.1 | 36.1 | Brapa_ESTC043107, 16933 |
| At3g53720 | ATCHX20 (CATION/H+ EXCHANGER 20); monovalent cation:proton antiporter | 1.5 | 4.1 | 1.7 | 1.3 | Brapa_ESTC043715 |
| At1g05580 | ATCHX23 (CATION/H+ EXCHANGER 23); monovalent cation:proton antiporter | 2.5 | 2.0 | 11.5 | 3.1 | Brapa_ESTC029030 |
| At5g58460 | ATCHX25 (cation/hydrogen exchanger 25); monovalent cation:proton antiporter | -1.4 | -1.7 | 16.6 | 1.5 | Brapa_ESTC046409 |
| At5g01690 | ATCHX27 (cation/hydrogen exchanger 27); monovalent cation:proton antiporter | -1.3 | -1.0 | 11.7 | 1.6 | Brapa_ESTC032809, 20594, 27697 |
| At3g52080 | CHX28 (cation/hydrogen exchanger 28); monovalent cation:proton antiporter | -1.4 | -1.3 | 9.5 | 1.2 | Brapa_ESTC043562 |
| At1g03550 | Secretory carrier membrane protein (SCAMP) family protein | 1.0 | -1.6 | 14.8 | 1.4 | Brapa_ESTC039356 |
| At1g73220 | Sugar transporter family protein | -1.5 | -4.1 | 12.5 | 1.0 | Brapa_ESTC019715, 17048 |
| At4g04760 | Sugar transporter family protein | 9.9 | 56.4 | 4.8 | 58.0 | Brapa_ESTC017448, 26987, 31219, 50081, 49717, 50082 |
| At4g02050 | Putative sugar transporter | 1.3 | 15.4 | 11.3 | 21.9 | Brapa_ESTC016928 |
| At3g55100 | ABC transporter family protein | 5.9 | 260.1 | 268.2 | 567.7 | Brapa_ESTC007807 |
| At4g15236 | ABC transporter family protein | 1.2 | -1.6 | 3.1 | -1.7 | Brapa_ESTC036192 |
| At2g25600 | SPIK (SHAKER POLLEN INWARD K+ CHANNEL); cyclic nucleotide binding | 2.2 | 3.3 | 51.8 | 6.5 | Brapa_ESTC041235 |
| At5g13670 | Nodulin MtN21 family protein | 18.8 | 164.2 | 17.4 | 36.3 | Brapa_ESTC027159 |
| At5g47470 | Nodulin MtN21 family protein | 1.0 | -1.2 | 27.4 | 2.0 | Brapa_ESTC021062 |
| At1g50310 | Monosaccharide transporter (STP9) | -1.4 | 2.0 | 114.7 | 76.8 | Brapa_ESTC025843, 34265, 25541 |
| At2g33670 | MLO5 (MILDEW RESISTANCE LOCUS O 5) | 1.1 | 1.5 | 49.8 | 5.9 | Brapa_ESTC041638 |
| At1g31885 | Major intrinsic family protein / MIP family protein | 102.3 | 348.6 | 1.3 | 242.4 | Brapa_ESTC011581 |
| At1g52580 | Rhomboid family protein | 1.2 | -1.6 | 22.6 | 2.2 | Brapa_ESTC019256, 26010, 40951, 28362 |
| At1g71680 | Amino acid permease | -1.4 | -2.0 | 90.4 | 30.9 | Brapa_ESTC016920 |
| At4g35180 | LHT7 (LYS/HIS TRANSPORTER 7); Amino acid permease | 1.9 | 1.0 | 25.4 | 3.0 | Brapa_ESTC039136, 19279, 14834, 09336, 30481, 26048 |
| At1g31885 | Transporter | 5.8 | 35.2 | -2.0 | 76.5 | Brapa_ESTC031691 |
| At5g64560 | Magnesium transporter CorA-like family protein (MRS2-2) | 2.3 | 8.3 | -1.6 | 6.2 | Brapa_ESTC020685, 20255, 46558 |
